# Identifying protein complexes directly from high-throughput TAP data with Markov random fields

**DOI:** 10.1186/1471-2105-8-482

**Published:** 2007-12-19

**Authors:** Wasinee Rungsarityotin, Roland Krause, Arno Schödl, Alexander Schliep

**Affiliations:** 1 Max Planck Institute for Molecular Genetics, Department of Computational Molecular Biology, Ihnestr. 73, D-14195 Berlin, Germany; 2 Max Planck Institute for Infection Biology, Department of Cellular Microbiology, Charitéplatz 1, D-10117 Berlin, Germany; 3 Think-cell software, Invalidenstr. 43, D-10115 Berlin, Germany

## Abstract

**Background:**

Predicting protein complexes from experimental data remains a challenge due to limited resolution and stochastic errors of high-throughput methods. Current algorithms to reconstruct the complexes typically rely on a two-step process. First, they construct an interaction graph from the data, predominantly using heuristics, and subsequently cluster its vertices to identify protein complexes.

**Results:**

We propose a model-based identification of protein complexes directly from the experimental observations. Our model of protein complexes based on Markov random fields explicitly incorporates false negative and false positive errors and exhibits a high robustness to noise. A model-based quality score for the resulting clusters allows us to identify reliable predictions in the complete data set. Comparisons with prior work on reference data sets shows favorable results, particularly for larger unfiltered data sets. Additional information on predictions, including the source code under the GNU Public License can be found at http://algorithmics.molgen.mpg.de/Static/Supplements/ProteinComplexes.

**Conclusion:**

We can identify complexes in the data obtained from high-throughput experiments without prior elimination of proteins or weak interactions. The few parameters of our model, which does not rely on heuristics, can be estimated using maximum likelihood without a reference data set. This is particularly important for protein complex studies in organisms that do not have an established reference frame of known protein complexes.

## Background

Recent advances in proteomic technologies allow comprehensive investigations of protein-protein interactions on a genomic scale. Interacting proteins provide detailed information on basic biomolecular mechanisms and are a valuable tool in the exploration of cellular life. Protein complexes are physical entities that are formed by stable associations of several proteins to perform a common, often complex function; in fact most of the basic cellular processes such as transcription, translation or cell cycle control are carried out by protein complexes. The goal of our work is to identify protein complexes directly from experimental results obtained from co-immunoprecipitation techniques, in particular the important tandem affinity purification approach (TAP) [1]. TAP employs a fusion protein carrying an affinity tag that is used to bind the protein to a matrix; subsequent washing and cleavage of the tag allows for obtaining the complexes under almost native conditions. The identification of the mixture of different proteins is usually carried out by mass spectrometry. Genome wide screens using TAP are available for the yeast *Saccharomyces cerevisiae *[2,3].

In prior approaches for predicting protein complexes, the experimental observations had to be condensed into a protein interaction graph. A protein-protein interaction graph is an undirected graph *G *= (*V*, *E*) where *V *is a set of nodes representing proteins and *E *is a set of edges. An edge indicates, depending on the particular model, either a physical interaction or protein complex co-membership of two proteins and may be weighted to designate interaction probability. All approaches that use an unweighted (e.g., thresholded) interaction graph as an intermediate step suffer from the problem that the uncertainty contained in the observation is no longer represented in the interaction graph, and cannot be properly accounted for when computing the clustering.

Moreover, most existing techniques for predicting protein complexes rely on heuristics for further analysis of the protein interaction graph. Often several parameters have to be chosen, usually with very little guidance from theory. Instead, parameters are optimized on benchmark data sets [2,4,5] and thus depend on the existence of such data sets for successful prediction. Other, more stringent algorithms suffer from the requirement of having an absolute measure of an interaction as input [6,7].

In contrast to previous methods that rely on constructing an intermediate interaction graph, our model-based approach uses the experimental measurements directly, which should provide a more rigorous framework for protein-protein interaction analysis. Our probabilistic model explicitly and quantitatively states the assumptions about how protein interactions are exposed by the experimental technique. A suitable algorithm then uses this model to subsequently compute a clustering.

For this work, we focus on partitioning proteins into complexes. Furthermore, any pair of proteins is assumed to either interact or not, independent of the context of other proteins in which it appears. As a consequence, clusters never overlap and each protein is assigned only to a single cluster. Several proteins are known to be part of more than one protein complex. While the problem is biologically relevant, only few proteins are *bona fide *members of many complexes [8] and even more complex methods such as used by Gavin *et al. *identify largely non-overlapping solutions (cores) as basic, reliable elements [2].

Our work is inspired by an approach for evaluating protein-protein interaction from TAP data by Gilchrist *et al. *[9] that calculated maximum-likelihood estimates of false negative error rate, false positive error rate and prior probability of interaction, but which cannot compute protein complexes. Our model uses their observation model, but we also compute likely protein complexes along with maximum-likelihood estimates of error rates.

**Figure 1:**
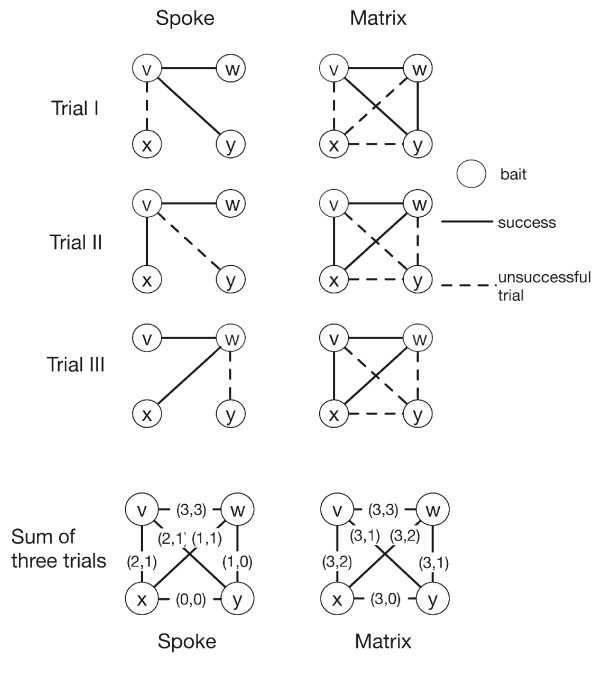
**Observational model for three hypothetical trials**. Two proteins are connected by an edge if their interaction is tested by a trial. The last row shows the observation from the three trials in their (*t*, *s*) values assuming the spoke and matrix model. The spoke model counts pairwise interactions only between bait and preys. The matrix model counts all pairs of proteins in a purification. It follows that the matrix model creates more unsuccessful trials.

There are two extreme cases in the interpretation of purification experiments. One is the minimally connected spoke model, which converts the purification results into pairwise interactions between bait and preys only. The other is the maximally connected matrix model, which assumes all proteins to be connected to all others in a given purification [5]. While the real topology of the set of proteins must lie between these two extremes, most previous works focused on the spoke model of interaction [5,9]. From a sampling perspective, each purification given a certain bait protein and its preys can be seen as a trial to gather information on which of these proteins interact. For illustration, we use the example given in [9] for a scenario involving four proteins *v*, *w*, *x*, *y *(Figure 1). Assuming the spoke model and choosing *v *as a bait protein, we can view this experiment as a trial to observe three interactions between *v *and the proteins *w*, *x*, *y*. In repeating this experiment, we would have a second trial to observe these three interactions. A third experiment, now using protein *w *as a bait, provides a third trial to observe an interaction between *v *and *w*, as well as the first trial to observe an interaction between *w *and proteins *x *or *y*. Combining these three experiments, we have three trials for observing an interaction between *v *and *w*, two trials for observing an interaction between *v *and *x *and no trials for observing an interaction between *x *and *y *(see Figure 1). We define *t *as the number of trials in which we might observe an interaction between two proteins. For example, from these three experiments and assuming the spoke model, *t *is equal to 3, 2, 1 and 0 for the protein pairs (*v*, *w*), (*v*, *x*), (*w*, *x*) and (*x*, *y*), respectively. Assuming the matrix model, *t *is equal to 3 for all protein pairs. Notice that in the matrix model the pair (*x*, *y*) is tested 3 times while in the spoke model this pair is not tested at all (*t *= 0).

However, in each trial we may or may not observe an interaction. Consequently, we define *s *(for success) as the number of experiments in which we observe two proteins to interact (0 ≤ *s *≤ *t*). In Figure 1, using the spoke and matrix model respectively, we illustrate how the experimental results from the three experiments can be summarized as a set of observation (*t*, *s*) values for each possible pair of proteins, which form the basis of our observation. After the transformation, an interaction probability can be calculated using a statistical model of interaction [9]. In this work, we will directly use these counts to build a Markov random field (MRF) model of protein complexes and estimate the number of clusters as well as false negative and false positive rates.

Markov random fields have been successfully applied as a probabilistic model in many research areas, e.g. as a model for image segmentation in image processing [10]. In biological network analysis, MRF were used to model protein-protein interaction networks to predict protein functions of unknown proteins from proteins with known functions [11]. They were also used to discover molecular pathways, for example by combining an MRF model of the protein-interaction graph with gene expression data [12]. Our model differs from these previous works in that we use MRFs to model protein complexes without an intermediate interaction graph and model the observational error directly. We incorporate the observation error into the formulation of the model and apply Mean Field Annealing to estimate the assignment of proteins to complexes.

For estimating protein-protein interaction graphs, several protein-protein interaction databases are available, in particular for the yeast proteome. They mostly rely on data from the yeast two-hybrid system [13,14] and the tandem affinity purification-mass spectrometry analysis of protein complexes [2,3,15] and individual studies that focus on particular aspects [16,17]. Creating a protein interaction network from high-throughput experiments is difficult due to high error rates. Therefore, with present techniques, the resulting networks are often not accurate [18]. Current approaches merge the results of different types of experiments such as two-hybrid systems, mRNA co-expression and co-immunoprecipitation such as TAP-MS. In that, much information on experimental details is lost, which we would like to exploit. We therefore focus on TAP-MS results as experimental data source, which outperforms other techniques in accuracy and coverage in yeast [19,20].

In the following, we introduce two computational methods previously described that predict protein complexes given pairwise protein-protein interactions, which are most comparable and relevant to our approach [5,21]. Molecular Complex Detection (MCODE) [5] detects densely connected regions in a protein-interaction graph. First it assigns a weight to each vertex computed by its local neighborhood density, a measure related to a clustering coefficient of a vertex. Then, starting from a vertex with the highest density, it recursively expands a cluster by including neighboring vertices whose vertex weights are above a given threshold. Vertices with weights lower than the threshold are not considered by MCODE. The method can retrieve overlapping complexes, but in practice many proteins are left unassigned by MCODE.

Another popular approach applies the Markov Clustering algorithm (MCL) [21,22] to predict protein complexes, usually after low quality interactions are removed from the data set. In the application of MCL used by Krogan *et al. *[3], first several machine learning techniques are combined to model interaction probability from mass spectrometry results. In the next step, an intermediate interaction graph is generated by removing interactions with probability lower than a given threshold. MCL is then applied on the resulting graph to predict complexes. MCL simulates a flow on the graph by calculating powers of the transition matrix associated with the interaction graph. Its two parameters are the expansion and inflation values, the latter influencing the number of clusters. MCL produces non-overlapping clusters.

Following the statistical approach to model protein interaction [9], we consider each purification experiment to be an independent set of observations of the interaction or non-interaction of proteins. We model the assignment of proteins to complexes as a Markov random field (MRF). The model incorporates the observational error as false positive and false negative error rates, which are assumed to be identical for all purifications. The cluster assignment is computed using Mean Field Annealing (MFA), which requires two input parameters, the number of clusters *K *and the log-ratio of error rates *ψ*. We systematically estimate both the cluster assignment of proteins and the false positive and false negative error rates using maximum likelihood. We explore both spoke and matrix model and compare the solutions to other published solution of protein complexes. Data sets and the detailed description of methods can be found in the Methods section.

## Results

### Performance on simulated data

**Figure 2:**
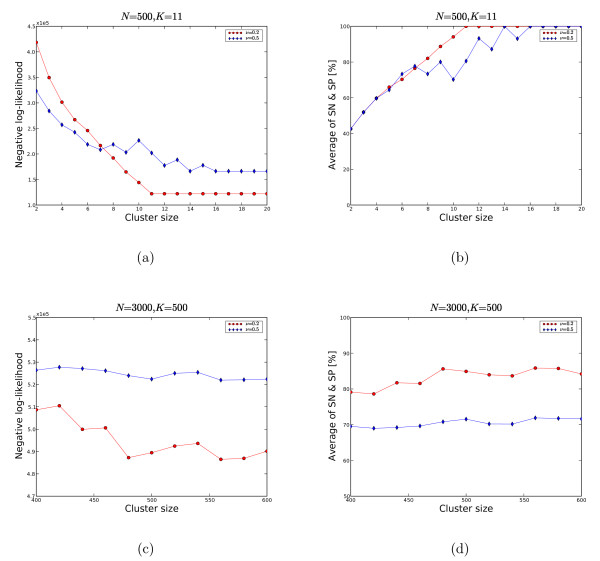
**MRF on simulated data**. We tested two sets of simulated data: (1) *N *= 500, *K *= 11 and (2) *N *= 3000, *K *= 500 and the false positive rate *φ *is set to 0.005 and the false negative rates *ν *is 0.2 or 0.5. With *ν *= 0.2 (2(a), 2(b)), MRF can recover the true clustering with the minimum negative log-likelihood which is taken on for 11 clusters. Notice that any more clusters do not reduce the cost any further; additional clusters simply remain empty. For *ν *= 0.5, the accuracy is worse and needs more empty clusters to reach convergence. In 2(c) and 2(d) the convergence rate fluctuates more.

To test convergence of our algorithm irrespective of the starting point, we first ran it on simulated data. We created the data from a set of *N *nodes, which we randomly assigned to *K *clusters. The number of trials *t *was the same for each pair of nodes, with the number of successes *s *reflecting the specified values of the false negative rate *ν *and the false positive rate *φ*. We ran the algorithm multiple times with different random starting points and initial values for *ψ*. We tested the algorithm on two problem sizes: (1) a small size *N *= 500, *K *= 11 and (2) a large size *N *= 3000, *K *= 500. We set *φ *to be 0.005, which is similar to the MIPS data (Table 1) and tested two values of *ν*: 0.2 and 0.5 [23]. We computed the average minimum cost at a given number of clusters, as shown in Figures 2(a) and 2(c). Figures 2(b) and 2(d) depict the quality of our solution as the geometric average of sensitivity (SN) and specificity (SP).

**Table 1:** Maximum likelihood solution for the spoke model (*ψ *= 3.5) and the matrix model (*ν *= 10.0). We choose the number of clusters that maximizes the likelihood by searching over a range of values of *K*. The estimated the false negative rate is denoted by ν* and the estimated false positive rate by φ*. For comparison we show the error estimates based on the MIPS complexes, *ν*_*MIPS *_and *φ*_*MIPS*_, restricted to proteins with MIPS annotation. See also Table 2.

Dataset		*K*	ν*	φ*	*ν*_*MIPS*_	*φ*_*MIPS*_
*Gavin02*	Spoke model	393	0.423	1.3 × 10^-3^	0.598	6.5 × 10^-3^
	Matrix model	310	0.752	1.7 × 10^-3^	0.717	5.2 × 10^-3^
*Gavin06*	Spoke model	698	0.547	2.4 × 10^-3^	0.637	8.3 × 10^-3^
	Matrix model	550	0.807	2.7 × 10^-3^	0.901	6.4 × 10^-3^

For the small problem size, Figures 2(a) and 2(b) show that the algorithm converges to the correct solution, with correct cluster assignments as well as correct estimates of the model parameters, *ν *and *φ*. With the high false negative rate of 0.5, the algorithm needs more clusters, some of which remain empty, to arrive at the correct solution. For the larger problem size of *K *= 500, we searched all *K *from 400 to 600 in steps of 20. The estimate of the error rate is approximately correct and the likelihood takes a minimum around *K *= 480 (see Figure 2(c)), but we only come close to the correct cluster assignment, with about 85% of all pairs correctly identified.

Ideally, we can estimate the number of clusters *K *from the likelihood of the solution for each *K*. When increasing *K*, the likelihood of the computed solution is increasing as long as the added clusters are used for a better cluster assignment of proteins. The likelihood is going to reach its maximum if all proteins are correctly assigned. Any additional clusters will remain empty, and the likelihood will increase no further (Figure 2(a)). In reality, with large problem sizes, the solution does not converge to the optimum cluster assignment, in particular when noise is present. The flattening of the likelihood however indicates that the correct number of clusters has been reached (Figure 2(c)).

### Clustering of data sets obtained in high-throughput experiments

For clustering proteins, we compute clusters for two types of observation models: the spoke model and the matrix model of protein interactions. To find a maximum likelihood solution, we first use a large number of clusters to search for a *ψ *maximizing the likelihood. For that *ψ*, we then run the optimization for different cluster sizes. We do three runs per cluster size to control for influences of the optimization starting point, and use the one with the highest likelihood. The maximum likelihood solutions are shown in Table 1. The estimated false positive rate φ* of our clustering solution is on the order of 10^-3 ^agrees with previously published results [9]. Note that by our definition, the false positive rate is the fraction of interactions observed between distinct complexes of the model divided by the number of all tested interactions between distinct complexes, which are present in the observation. For example, given our cluster solution for the spoke model, there are approximately 6 million trials between distinct complexes (2760 proteins) and among them, we observe about 14100 false positives. The number of trials within complexes is much smaller, about 14000 trials in total, but only about half of them are observed, resulting in a false negative rate of approximately 0.5. Based on the experimentally observed interactions, about 70% are false positive. However, this is not the definition of the error rates used by our model.

We have also calculated the error rates based on the MIPS data [23]. The false negative rate is very close to the one we estimated for our solution. The false positive rate is still of the same magnitude, but 2 to 5 times larger than the false positive rate computed for our solution. The decisions underlying the manually curated MIPS dataset were similarly conservative in assigning proteins to the same cluster as our algorithm. We discuss a method to distinguish reliable from less reliable clusters in our solution later. False positive rates in TAP-MS experiments are much lower than for other experimental techniques as has been reported earlier [19,20].

The approach presented here does not rely on a benchmark set. However, to evaluate the performance of the algorithm to extract relevant information from high-throughput data sets we compared it to the results of other algorithms (MCL, MCODE) and the protein complexes accompanying publications of the data sets. We use two data sets, *Gavin02 *and *Gavin06 *[2,15], to compare the results to earlier studies. The first data set was used in previous works to benchmark the predictions [24] and is basically a subset of the second. See Table 2 and the Methods section for the description of the data sets.

**Table 2:** Data set and results sizes. MCL and MRF consider the same number of proteins: all proteins in the experiments. However, their clustering solutions are different; MCL will produce more singletons than MRF.

Dataset	Num. Proteins		MCL	MRF	MCODE	Gavin06 (all)	Gavin06 (core)
*Gavin02*	1390	Proteins clustered	1390	1390	112	-	-
		with MIPS	494	494	53	-	-
		with Reguly	136	136	20	-	-
*Gavin06*	2760	Proteins clustered	2760	2760	243	1488	1147
		with MIPS	819	819	141	633	492
		with Reguly	520	520	120	429	336

Because MCL and MCODE require an interaction graph as input we construct one using a spoke model for each data sets. MCL accepts both weighted and unweighted graphs as an input. For the weighted interaction graph, we compute the interaction probability using the statistical model in [9] without a threshold.

**Figure 3:**
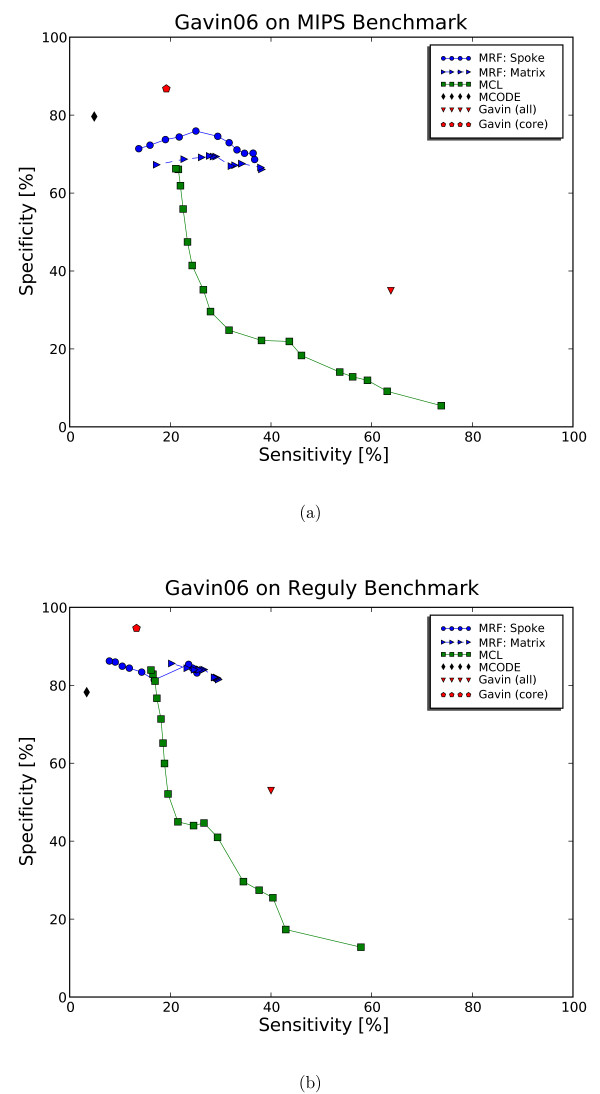
**Comparison of sensitivity and specificity for all clustering solutions on Gavin06**. Only proteins with annotation from MIPS (a) and Reguly (b) are considered. The curve for MRF is generated as we filter out clusters with high observed errors. The curve for MCL is generated for different inflation parameters, [1.2, 0.2, 5.0], which are recommended by the MCL program. Highly specific solutions are the MCODE and the *Gavin06 *(core) solutions with show low sensitivity due to many proteins left unassigned. MRF maintains better sensitivity while losing only a few percent in specificity. In this respect, MRF performs better than MCL because it maintains high specificity without losing sensitivity.

To set the inflation parameter for MCL, we find that the optimal setting as published in [24] is suitable for the smaller data set (*Gavin02*), but yields a biologically implausible small number of clusters for the larger *Gavin06 *data set. Therefore, we have explored several inflation parameters from the recommended range of 1.1 to 5.0. We found the inflation parameter of 3.0 to result in a number of clusters containing more than 2 proteins, which is close to the published number of 487 complexes [2]. The trade-off in sensitivity and specificity from exploring the inflation parameters is shown in Figure 3. We summarize the parameter setting for all three algorithms in Table 3. For comparison of the clustering algorithms, we compare the performance measures to evaluate the clustering solutions for the MIPS and Reguly data sets [23,25]. We compare these measures for clustering and random complexes and observe good separation. For the evaluation, we do not consider singletons as valid clusters and exclude them from the distribution of cluster sizes, see Table 4 and Table 5. We summarize the measurements in Table 6 for the *Gavin02 *data set and the *Gavin06 *data set.

**Table 3:** Parameter settings for MCL, MRF and MCODE.

Dataset	MCL	MCL with interaction prob. [9]	MRF	MCODE
*Gavin02*	From [24]	Inflation = 1.8	*ψ *= 3.5	From [24]
Spoke model	Inflation = 1.8	*ν *= 0.346	Maximum likelihood	Node score percentage = 0.0
		*φ *= 1.07 × 10^-3^		Complex fluff = 0.2
		*ρ *= 1.88 × 10^-3^		Depth = 100
*Gavin06*	Inflation = 3.0	Inflation = 3.0	*ψ *= 3.5	From [24]
Spoke model		*ν *= 0.407	Maximum likelihood	Node score percentage = 0.0
		*φ *= 1.35 × 10^-3^		Complex fluff = 0.2
		*ρ *= 3.89 × 10^-3^		Depth = 100
*Gavin06*	-	-	*ψ *= 10.0	-
Matrix model			Maximum likelihood	

**Table 4:** Distribution of cluster sizes for the *Gavin02 *data

	MCL	MCL with inter. prob.	MRF (spoke)	MRF (matrix)	MCODE
Num. of clusters	351	352	393	310	24
Num. of singletons	177	178	226	79	0
Size ≥ 2	174	174	167	231	24
Mean	6.97	6.97	6.97	5.67	4.67
Median	4	5	5	2	4
1st quantile	3	3	2	2	3
3rd quantile	8	8	10	6	6
90%	15	14	14	13	7
99%	42	40	34	36	9
Largest cluster	51	45	36	44	11

**Table 5:** Distribution of cluster sizes for the *Gavin06 *data.

	MCL	MCL with inter. prob.	MRF	MRF (matrix)	Gavin06 (all)	Gavin06 (core)	MCODE
Num. of clusters	781	732	698	550	487	477	55
Num. singletons	331	269	4	2	0	55	0
Size ≥ 2	450	463	694	548	0	422	0
Mean	5.39	5.38	3.97	5.03	13.46	3.33	4.42
Median	2	3	2	3	9	2	4
1st quantile	2	2	2	3	4	2	3
3rd quantile	4	4	4	5	18	4	5
90%	8	7	7	8	33	6	7
99%	36	29	32	31	66	12	16
Largest cluster	561	607	65	49	96	23	16

**Table 6:** Clustering performance of MCODE, MCL and MRF: comparison with the MIPS annotations. We use all proteins in the experiment with annotation.

Dataset		MCODE	MCL	MCL with inter. prob.	MRF (spoke)	MRF (matrix)
*Gavin02*	CO	29.0	61.5	62.6	64.4	**66.4**
	PPV	**73.6**	71.3	71.7	73.5	66.9
	*Acc*	46.2	66.2	67.0	**68.8**	66.6
	All pairs					
	SN	2.3	68.6	**68.9**	66.7	62.6
	SP	**92.5**	78.7	82.4	87.9	64.7
	Geo. average	14.7	73.0	75.4	**76.6**	63.6
*Gavin06*	CO	33.7	64.0	65.7	66.0	**67.7**
	PPV	**79.0**	62.6	68.6	70.4	67.3
	*Acc*	51.6	63.3	67.2	**68.2**	67.5
	All pairs					
	SN	4.9	44.1	**44.7**	37.2	38.2
	SP	**79.6**	18.0	22.5	70.0	66.1
	Geo. average	19.7	28.2	31.7	**51.0**	50.2

For each data set, we use the set of annotated and clustered proteins for the evaluation. Note that this can lower sensitivity and complex-coverage in the results of algorithms such as MCODE that leave proteins unassigned. The results are shown in Table 6 and the ROC curves in Figure 3. As expected, we find clustering solutions of MCODE to have low sensitivity (low complex-coverage) and high specificity because it assigns only few proteins and ignores the majority of proteins present in the experiment. We set the parameters of MCODE as described by Brohée and van Helden [24]. When we changed the setting of MCODE to include more clusters and assign more proteins, we significantly lose accuracy in all measures.

### Testing

To extract relevant information from our clusters, we compare the results to the MIPS and Reguly data sets. We apply two evaluation procedures: one based on a set of benchmark procedures recently introduced by Brohée and van Helden [24] and the other based on the pair-wise comparisons of proteins.

Comparing a clustering result with annotated complexes using the evaluation procedure of Brohée and van Helden [24] starts with building a contingency table. With *n *complexes and *m *clusters, the contingency table *T *is an *n × m *matrix whose entry *T*_*ij *_is the number of proteins in common to the *i*th complex and the *j*th cluster. Given a contingency table *T*, overall accuracy and separation value can be computed to measure the correspondence between clustering result and the annotated complexes [24]. The separation measure yields undesirable effects when the reference data set contains overlapping complexes because according to its definition [24], a good match of a cluster to more than one complex will result in a low separation value. This situation arises for the MIPS and Reguly benchmark, which are overlapping, while the computed results of MCL and MRF are not. Furthermore, when matching the reference data set to itself, we found that its separation value can be less than that of some clustering solutions. For these reasons, we do not apply the separation measure. The definitions related to benchmarks are summarized in the Methods section.

#### Quality of clusters

In any given solution, some clusters will have more support from the observation than other clusters. Support for a cluster is high if proteins in this clusters are less likely to be part of false positive or false negative observations. So we can compute a cluster quality metric as the difference between the *actual *number of false positives and false negatives and their *expected *number, based on the number of trials involving proteins of this cluster. Let *Q*_*i *_be the cluster assignment for protein *i*, ν * the estimated false negative rate and φ * the estimated false positive rate. Then the difference between actual and expected errors *E*(*k*) for each cluster *k *is



where Efn(k)=ν∗∑(i,j):Qi=Qj=ktij
 MathType@MTEF@5@5@+=feaagaart1ev2aaatCvAUfKttLearuWrP9MDH5MBPbIqV92AaeXatLxBI9gBaebbnrfifHhDYfgasaacPC6xNi=xH8viVGI8Gi=hEeeu0xXdbba9frFj0xb9qqpG0dXdb9aspeI8k8fiI+fsY=rqGqVepae9pg0db9vqaiVgFr0xfr=xfr=xc9adbaqaaeGacaGaaiaabeqaaeqabiWaaaGcbaGaemyrau0aaSbaaSqaaiabdAgaMjabd6gaUbqabaGccqGGOaakcqWGRbWAcqGGPaqkcqGH9aqpiiGacqWF9oGBdaahaaWcbeqaaiabgEHiQaaakmaaqafabaGaemiDaq3aaSbaaSqaaiabdMgaPjabdQgaQbqabaaabaGaeiikaGIaemyAaKMaeiilaWIaemOAaOMaeiykaKIaeiOoaOJaemyuae1aaSbaaWqaaiabdMgaPbqabaWccqGH9aqpcqWGrbqudaWgaaadbaGaemOAaOgabeaaliabg2da9iabdUgaRbqab0GaeyyeIuoaaaa@4C6E@ and Efp(k)=ϕ∗∑(i,j):Qi≠Qj=ktij
 MathType@MTEF@5@5@+=feaagaart1ev2aaatCvAUfKttLearuWrP9MDH5MBPbIqV92AaeXatLxBI9gBaebbnrfifHhDYfgasaacPC6xNi=xH8viVGI8Gi=hEeeu0xXdbba9frFj0xb9qqpG0dXdb9aspeI8k8fiI+fsY=rqGqVepae9pg0db9vqaiVgFr0xfr=xfr=xc9adbaqaaeGacaGaaiaabeqaaeqabiWaaaGcbaGaemyrau0aaSbaaSqaaiabdAgaMjabdchaWbqabaGccqGGOaakcqWGRbWAcqGGPaqkcqGH9aqpiiGacqWFvpGzdaahaaWcbeqaaiabgEHiQaaakmaaqafabaGaemiDaq3aaSbaaSqaaiabdMgaPjabdQgaQbqabaaabaGaeiikaGIaemyAaKMaeiilaWIaemOAaOMaeiykaKIaeiOoaOJaemyuae1aaSbaaWqaaiabdMgaPbqabaWccqGHGjsUcqWGrbqudaWgaaadbaGaemOAaOgabeaaliabg2da9iabdUgaRbqab0GaeyyeIuoaaaa@4D43@.

**Figure 4:**
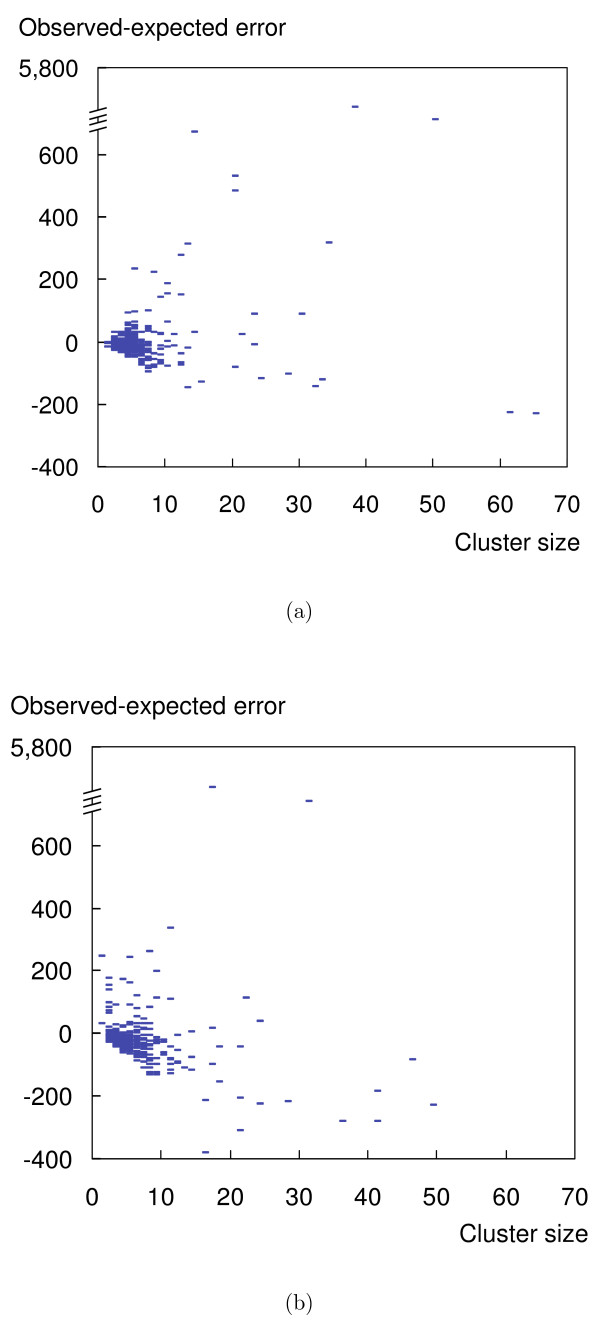
**Cluster quality**. The distribution of the quality of clusters as predicted by MRF. Note that negative values for the quality score indicate that a cluster is observed better supported by the data than expected. The zero-line indicates that the observed error corresponds to the expectation. (a) shows that most predicted clusters fit the model for the spoke model. Outliers with high error are points on the top of the figure; the clusters contain largely artifacts. (b): Also for the matrix model, MFA is robust against the high false negative rate. For the list of clusters, refer to the supplementary material.

Figure 4 shows the distribution of *E*(*k*) for the spoke and matrix models. The score is positive for some clusters and negative for others, with the mode around zero. So rather than giving an absolute measure of quality for the whole solution, the measure indicates, within a given solution, clusters with high confidence and those with low confidence. Figure 4 shows that there is no correlation between the score *E*(*k*) and cluster sizes. They also show that we have discovered quite reliable observations for some large clusters. MRF has also identified some outliers with extremely high error score; they consist of abundant proteins that are found unspecifically with many purifications, typically more than 50.

#### Complex-size distribution

**Figure 5:**
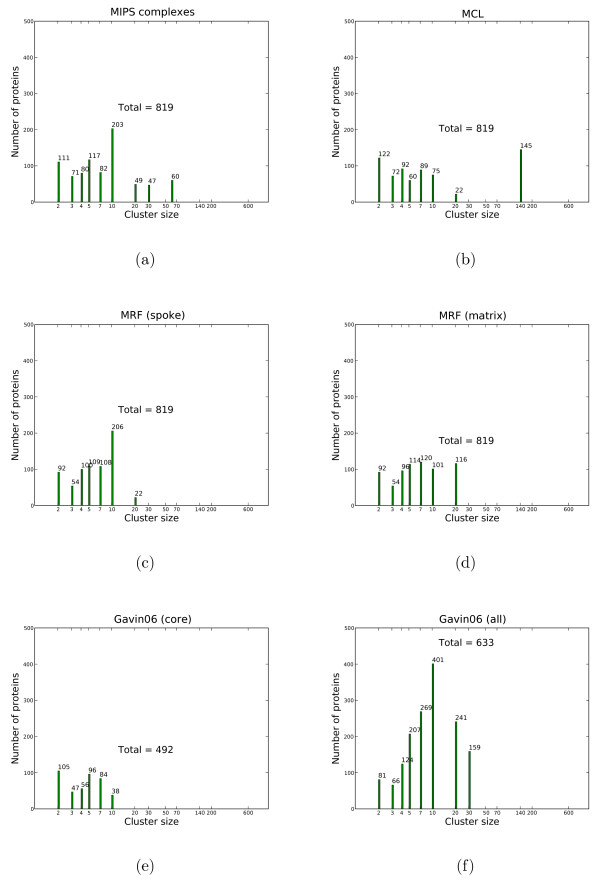
**Cluster sizes by MIPS proteins on the Gavin06 set**. The x-axis shows cluster sizes in log-scale. The y-axis shows the number of proteins in a cluster of certain size by proteins found in MIPS complexes. Note, that singletons and proteins not contained in the MIPS set are not considered. Each column also shows the total number of proteins. Cluster sizes are taken from either the primary data source – MIPS(a), Gavin06 (core) (e) and Gavin06 (all) (f) – or solutions obtained on the *Gavin06 *data set – MCL(b), MRF (spoke)(c), MRF (matrix) (d).

**Figure 6:**
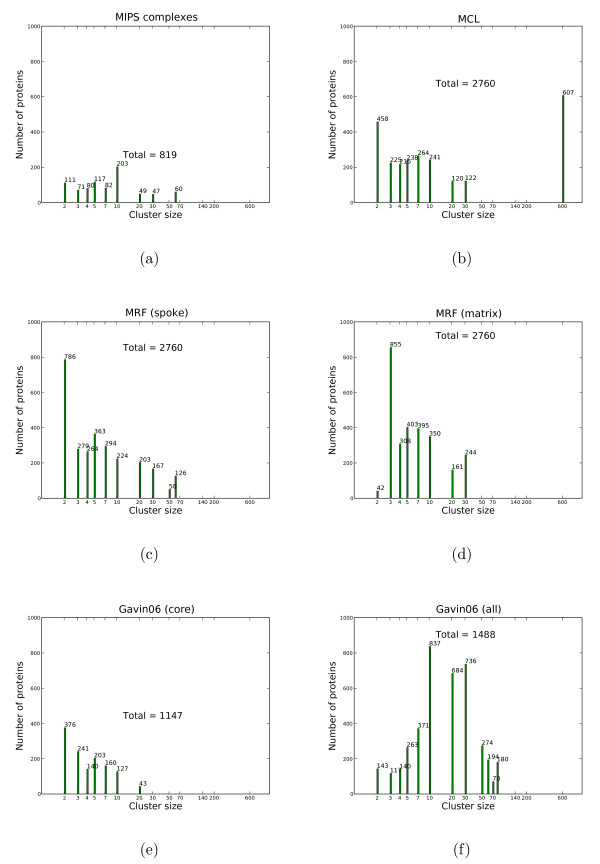
**Cluster sizes by all proteins of the Gavin06 set**. When considering all proteins, not only those contained in the MIPS set, all clustering solutions deviate substantially from the MIPS set's size distribution. A cluster from MCL with 607 proteins is a giant component which merges smaller MIPS complexes with many other proteins.

Principle properties and potential artifacts are visible in a simple plot of the population of proteins by cluster size (see Figures 5 and 6). In Figure 5, we only consider proteins with MIPS complexes assigned from the *Gavin06 *data set, ignoring singletons; this results in 819 proteins. For each clustering solution, we compute the cluster size distribution of MIPS proteins which have cluster assignments. It is worth to note that there is an absence of MIPS complexes in the range from 20 to 30. Obviously, the proteins in the largest complex of size 60 all correspond to a single complex (the ribosome), whereas the 60 proteins in clusters of size 12 correspond to 5 different clusters. In Figure 6, when considering all proteins, all clustering solutions substantially deviate from the MIPS size distribution. MCL has a large cluster containing 607 proteins, likely an artifact. The Gavin core set is only a subset and contains a substantial number of small elements and fewer complexes than the MIPS solution, prominently the mitochondrial ribosome and mediator complex. The larger, complete solution (Gavin06 (all)) contains few small clusters; although this solution contains larger clusters (size ≤ 50), they do not accurately map to larger complexes. In Figure 6, our MRF solution for the spoke model contains more clusters of size 2 than the matrix model, but otherwise both have similar size distribution with more small clusters than large ones.

### Cluster visualization

**Figure 7:**
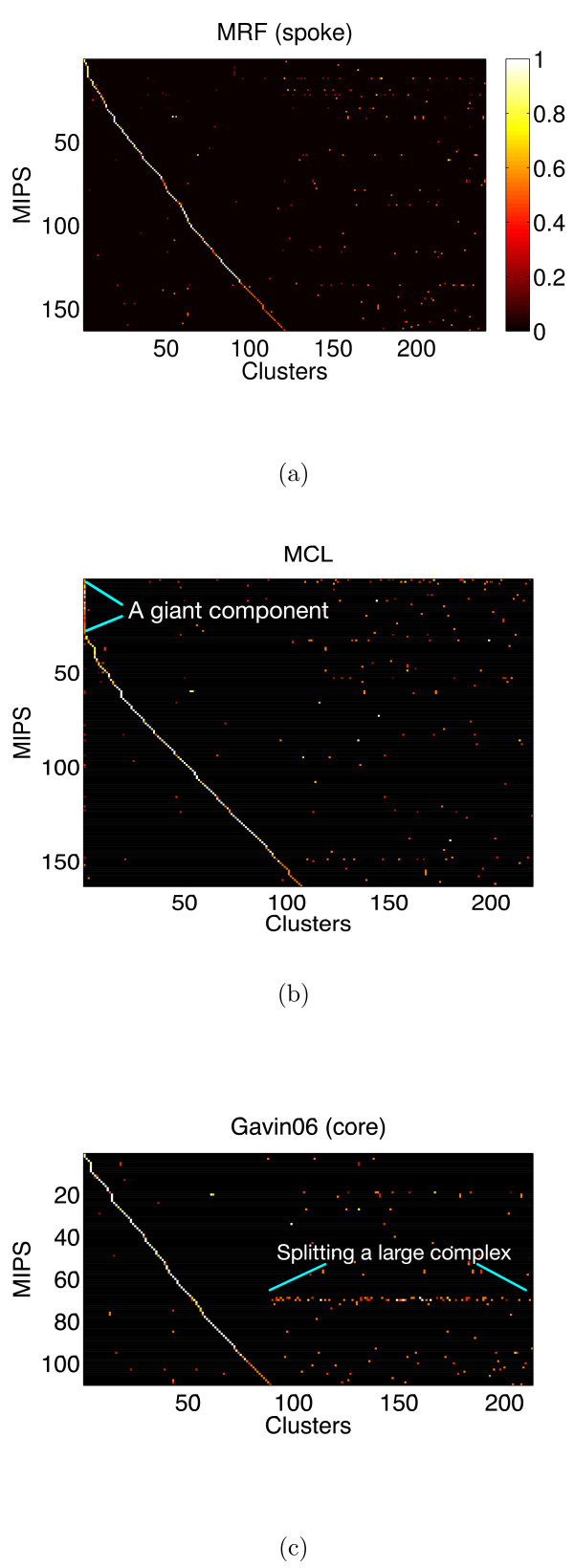
**Mapping to the MIPS complexes**. Visualization of the best mapping to the MIPS complexes on the *Gavin06 *data set. Figures are contingency tables where each cell is the Simpson coefficient with values from [0, 1]. We show three solutions from MRF (spoke) (a), MCL (b) and the core set of *Gavin06 *(c). Rows are MIPS complexes and columns are clusters obtained using the respective algorithm. The order of complexes and clusters differ between figures. Complexes without mapping to any cluster are not part of a table and likewise for clusters without mapping to any complex. Each figure has a different range of the x-axis and y-axis, because each solution has a different number of clusters mapping to a different subset of the MIPS complexes. The *Gavin06 *(core) solution maps to fewer complexes because it assigns fewer proteins.

For each clustering solution, we can visualize matches to the MIPS complexes by generating a contingency table whose rows are complexes and columns are clusters. For each cell in the table, we calculate the Simpson coefficient [4] and order the diagonal of the table by increasing matching sizes. Clusters without any matches to annotated complexes are not part of the table, neither are complexes without a match to any cluster. In Figure 7, we summarize the mapping of MRF (spoke model), MCL and the core Gavin06 solutions. For more visualization of other clustering solutions and mapping to the Reguly benchmark, refer to the supplementary material. We also visualize how well each solution maps to the complex-size distribution. For each clustering solution, we plot the histogram of cluster size distribution on the log-scale.

Figure 5 shows cluster sizes by proteins found in MIPS complexes, while Figure 6 uses all proteins. Note the largest cluster in the MCL solution, which contains a very diverse range of proteins, is likely to be an artifact.

### Examples

A positive evaluation of a clustering procedure by internal and by clustering indices does not necessarily mean that the results are useful and match a user's expectation. Above, we compared the results on a large scale, here we inspect the solutions in detail. When selecting biological examples, the MRF solution under the spoke model seems to produce better results for smaller complexes. Note for example the underrepresentation of size 2 complexes under the matrix model in Figure 5. (Table 5). The high false negative rate of the matrix model could imply that it is less capable than the spoke model. Nonetheless, it recovers meaningful clusters, showing that it is robust against such high error rate as can be seen in the benchmark in Figure 3.

Our MRF solution for the spoke model contains two largest complexes of size > 60 of presumably high quality. Contradicting the observation that the spoke model appears to produce better results for larger complexes (Figure 4), manual inspection suggests that these structures are not similar to complexes like the ribosome or the proteasome but a rather spurious collection of proteins that interact. The two largest clusters in the spoke model do not constitute known complexes and highlight a peculiar property of the TAP-MS data set. Apparently, high-quality results are sporadically obtained for rather well characterized proteins that seem to link very different pathways and cellular localizations. Although the two clusters have high quality with respect to the model, we find that there are not enough repetitions for these proteins and in practice we must interpret their interactions as of medium confidence only.

There is no general agreement in the field how a protein complex should be defined biochemically. Many factors – binding constants, protein concentration and localization, different purification protocols – lead to different associations of proteins into aggregates that we consider complexes. Moreover, paralogous proteins that lead to variant complexes complicate the distinction of similar complexes. Disagreeing solutions for protein complexes offered by different methods do not necessarily indicate that either solution is wrong. For some complexes, all methods compared in this context lead to the identical solutions, such as the Arp2/3 complex (MRF259) or the origin of replication complex (MRF567). These complexes that are similarly found by all methods generally receive good (negative) quality scores *E*(*k*) in our model, indicating that all methods work for simple cases.

For larger complexes that can be well studied such as the Proteasome, the results appear fairly consistent across the different solutions. The best MCL solution according to our benchmark only splits Pre8, an element of the 20S subunit, into a separate complex and assigns several components of the 19S subunit to a giant cluster together with many unrelated proteins. The MRF solution appears slightly superior to the predicted cores in Gavin *et al. *[2] in that no components of the 19S subunit are assigned to other elements. A complex that appears not represented well in our set are the RNA Polymerases, three complexes of 12–14 proteins that share 5 proteins. An ideal solution would either place all elements of the complexes into one or into three clusters. While the Gavin solution neatly separates the complexes, the MRF solution only places several elements of the RNA Polymerases into clusters of low quality. The high quality cluster containing most elements of RNAPII, the best characterized complex of the three by experimental data, is "contaminated" with specific members of the other two complexes. The MCL solution displays similar problems.

One solution to the clustering that we find superior in our results is complex 239 from the spoke model, consisting of Sol2, Ade16, Ade17, Ste23, Sol1, Rtt101, and Yol063c. Sol1 and Sol2 are not part of the same complex in the Gavin set of complexes or the MCL solution, and do not interact observably, but are homologues. The isoenzymes Ade16 and Ade17 are not part of the same complex in the solution in Gavin *et al. *[2] but can be assumed to have the same binding partners.

## Discussion

Before we discuss the details of the results, we would like to point out that MRF is essentially a parameter-free method. Although MFA requires two inputs, *ψ *and the number of clusters *K*, we provide a systematic way to estimate them using maximum likelihood. Methods such as MCODE require more parameters without a systematic way to select them other than trying out several values and comparing the results to benchmark data. If there is no such data set available, these methods cannot asses the quality of their solution, while the value of the likelihood function can be used for our MRF approach. MCL suffers from the same problem of parameter selection and essentially has three parameters, the expansion and inflation values and the number of clusters. So to choose a solution from MCL we must not only compare with the benchmark, but also decide if the number of clusters is biologically plausible. With regard to the number of predicted clusters, it is not surprising that MRF estimates higher number of clusters because it does not eliminate proteins prior to clustering, unlike other solutions [3].

Although we recommend the spoke model over the matrix model due to lower false negative rate, it is noteworthy that the solution of the matrix model is also biological meaningful when compared to the MIPS data set, although with slightly lower specificity than the spoke model (on the *Gavin06 *data set comparing to the MIPS data set). In reality, the model of interaction likely lies in between the two extremes. With regard to the quality of the clusters, we observe that almost all predicted clusters fit the model except some outliers that should not be regarded as complexes due to extremely high observed errors (shown as data points on the top of Figure 4(a) and 4(b)). Closer inspection reveals that they are clusters consisting mostly of proteins that are systematic contaminants; one would not assign them to any complex manually. By giving these "junk" clusters the worst quality score, MRF can separate them from the rest of other complexes. For MCL, there is no such indicator.

The performance of MCL and MRF on the *Gavin02 *data set is comparable as both achieve high accuracy. This is the result of the lower level of noise in the *Gavin02 *data, which was filtered for abundant proteins. Error modeling does not necessarily yield more accuracy. Note also the similar distribution of cluster sizes (see Table 4).

The performance gain from error modeling is more noticeable in the larger *Gavin06 *data set which is not filtered and likely contains more errors. The accuracy *Acc *is the average of the agreement of a cluster to a complex. It penalizes complexes that are split more than complexes that are merged. To see if complexes are merged, we have to consider at the all pairs comparison for high sensitivity with low specificity. Due to complexes merged in a giant component, MCL performs quite well on *Gavin06 *measured by the accuracy value, but not when we consider the all-pairs sensitivity (SN) and specificity (SP) and comparing to the MIPS data set. To avoid the giant component, the inflation parameter of MCL must be set to the maximum level recommended (inflation = 5.0) which reduces sensitivity (Figure 3). MRF in contrast can maintain high specificity without sacrificing sensitivity nor does it produce giant components. When comparing to highly specific solutions such as MCODE or *Gavin06 *(core) which assign fewer proteins, MRF loses only a few percent (less than 10%) in specificity, but gains about 30% in sensitivity and while clustering more proteins (Table 6).

In general, both MCL and MRF perform better when compared to the MIPS benchmark than to the Reguly data, with MRF performing better than MCL at matching both benchmark sets on the *Gavin06 *data set. Many complexes in the Reguly set are redundant and overlap, some even completely which no method possibly could recover from data. Hence, MCL and MRF will never be able to fully reconstruct the Reguly data set as they assume no overlap between protein complexes. On the MIPS complexes and based on all-pair comparison, MRF outperforms MCL. This indicates that in general the assumption of complex formation based on only pairwise interaction is a reasonable one producing few false positive errors. We can observe the giant component of the MCL solution in Figure Z(b) as the first column including several complexes. A perfect mapping would be displayed as a diagonal line with no off-diagonal entries. The results show that no solution provides the best mapping. Although the core solution of Gavin06 appears to have the cleanest mapping with few off-diagonal entries, it only contains 1147 proteins, while our solution includes all 2760 proteins. When comparing all solutions to the MIPS-size distribution in Figure F, we clearly see that MCL is particularly far off due to the giant component which assigns about 140 proteins from different MIPS complexes into the same cluster. The solution from MRF appears to be the closest match in this regard, although it still cannot reconstruct MIPS-complexes larger than 30. Other solutions also have the same problem; the Gavin06 (core) solution only maps to small complexes (size ≤ 20). MRF replaces large complexes by producing more smaller clusters than MIPS (size ≤ 10).

In summary, if the data has already been filtered as in the *Gavin02 *data set, MRF does not have an advantage over MCL and is computationally more expensive. When clustering large and noisy data set, the evaluation demonstrates that MRF is a more suitable method, due to its rigorous framework allowing parameter selection using maximum likelihood.

## Conclusion

We introduce a probabilistic model based on Markov random fields to identify protein complexes from data produced by large-scale purification experiments using tandem affinity purification and mass spectrometric identification. Unlike previous work, our model incorporates observational errors, which enables us to directly use the experimental data without requiring an intermediate interaction graph and without prior elimination of proteins from the sets. The assignment to clusters corresponding to protein complexes are computed with the Mean Field Annealing algorithm. Because there are proteins which cannot be well clustered, we also provide a model-based quality score for each predicted complex. Our method does not rely on heuristics, which is particular important for applications on protein complex studies in organisms that do not have an established reference frame. The model has two parameters, which are estimated from the experimental data using maximum likelihood, providing an elegant solution to the problem. Our results compare favorably on reference data sets, notably for the larger unfiltered data sets.

For future work, the hard assignments imposed by our model can be relaxed to capture overlapping complexes, but the model and minimization algorithm must be changed.

It would also be useful to have a quantitative estimate of the number of clusters *K*. One would need to trade off the increase in likelihood against the increase in the number of clusters, in effect finding the smallest number of clusters with almost maximal likelihood. One approach would be the minimum description length (MDL) criterion [26], a rigorous technique to assign costs to both observation likelihood as well as the number of clusters.

## Methods

### Data sets

#### Experimental data sets

We focused on the data published by Gavin *et al. *in 2002 (*Gavin02*) and 2006 (*Gavin06*) [2,15], which was found to be of high quality [19,20,27,28]. The experimental data sets were downloaded and parsed from the respective supplementary information that accompanied the original publication. We found further data sets [16,17] less suitable for benchmarking because the baits used in these studies were chosen to address specific questions. Hence they do not constitute representative samples. Another recent large scale screen in yeast did not publish the individual, repeated purifications, making it impossible to estimate the error model used here [3].

#### Protein complex annotation

• **MIPS**: The MIPS data set [23] is a standard data set for benchmarking methods for protein complex prediction. Note that it was largely created before high throughput data sets were published.

• **Reguly**: A manually curated dataset of protein-protein interactions encompasses protein complexes taken from the literature [25]. It is less selective than the MIPS benchmark, and has several complexes that overlap significantly due to differences between individual description of complexes.

### A model of protein complexes using Markov random fields

We assume that clusters do not overlap and each protein *i *belongs to exactly one cluster *Q*_*i *_∈ {1, ..., *K*}, where *K *is the number of clusters. We expect proteins in the same cluster to interact, and proteins belonging to different clusters not to interact. Our observation contains errors, with a false negative error rate *ν *that proteins of the same cluster are not observed to interact, and a false positive error rate *φ*, that proteins belonging to different clusters are observed to interact. These error rates are assumed to be the same for all interactions. We estimate them while computing the cluster assignments of proteins.

Define *S*_*ij *_to be the event that proteins *i *and *j *are observed to interact, and, likewise, *F*_*ij *_the event that they are not observed to interact. The probabilities of these two events, given *ν*, *φ *and *Q*, are

P[Sij|ν,ϕ,Q]={(1−ν):Qi=Qjϕ:Qi≠Qj,
 MathType@MTEF@5@5@+=feaagaart1ev2aaatCvAUfKttLearuWrP9MDH5MBPbIqV92AaeXatLxBI9gBaebbnrfifHhDYfgasaacPC6xNi=xI8qiVKYPFjYdHaVhbbf9v8qqaqFr0xc9vqFj0dXdbba91qpepeI8k8fiI+fsY=rqGqVepae9pg0db9vqaiVgFr0xfr=xfr=xc9adbaqaaeGacaGaaiaabeqaaeqabiWaaaGcbaacbeGae8huaaLaei4waSLaem4uam1aaSbaaSqaaiabdMgaPjabdQgaQbqabaGccqGG8baFiiGacqGF9oGBcqGGSaalcqGFvpGzcqGGSaalcqWGrbqucqGGDbqxcqGH9aqpdaGabaqaauaabiqaciaaaeaacqGGOaakcqaIXaqmcqGHsislcqGF9oGBcqGGPaqkcqGG6aGoaeaacqWGrbqudaWgaaWcbaGaemyAaKgabeaakiabg2da9iabdgfarnaaBaaaleaacqWGQbGAaeqaaaGcbaGae4x1dyMaeiOoaOdabaGaemyuae1aaSbaaSqaaiabdMgaPbqabaGccqGHGjsUcqWGrbqudaWgaaWcbaGaemOAaOgabeaakiabcYcaSaaaaiaawUhaaaaa@55A5@

and

P[Fij|ν,ϕ,Q]={ν:Qi=Qj(1−ϕ):Qi≠Qj.
 MathType@MTEF@5@5@+=feaagaart1ev2aaatCvAUfKttLearuWrP9MDH5MBPbIqV92AaeXatLxBI9gBaebbnrfifHhDYfgasaacPC6xNi=xI8qiVKYPFjYdHaVhbbf9v8qqaqFr0xc9vqFj0dXdbba91qpepeI8k8fiI+fsY=rqGqVepae9pg0db9vqaiVgFr0xfr=xfr=xc9adbaqaaeGacaGaaiaabeqaaeqabiWaaaGcbaacbeGae8huaaLaei4waSLaemOray0aaSbaaSqaaiabdMgaPjabdQgaQbqabaGccqGG8baFiiGacqGF9oGBcqGGSaalcqGFvpGzcqGGSaalcqWGrbqucqGGDbqxcqGH9aqpdaGabaqaauaabiqaciaaaeaacqGF9oGBcqGG6aGoaeaacqWGrbqudaWgaaWcbaGaemyAaKgabeaakiabg2da9iabdgfarnaaBaaaleaacqWGQbGAaeqaaaGcbaGaeiikaGIaeGymaeJaeyOeI0Iae4x1dyMaeiykaKIaeiOoaOdabaGaemyuae1aaSbaaSqaaiabdMgaPbqabaGccqGHGjsUcqWGrbqudaWgaaWcbaGaemOAaOgabeaakiabc6caUaaaaiaawUhaaaaa@558F@

A single purification experiment generates a set of such observations. Over the course of multiple purification experiments, each pair of proteins may be observed multiple times. We define *t*_*ij *_to be the total number of observations made for the protein pair (*i*, *j*), and *s*_*ij *_to be the number of these observations where an interaction was observed.

Then, given *ν*, *φ *and a configuration *Q*, the likelihood of observing a particular sequence of experimental outcomes (*t*_*ij*_, *s*_*ij*_) for all pairs (*i*, *j*) is

P[{(tij,sij)}|ν,ϕ,Q]=∏(i,j)P[Sij|ν,ϕ]sijP[Fij|ν,ϕ]tij−sij=∏(i,j):Qi=Qj(1−ν)sijν(tij−sij)∏(i,j):Qi≠Qjϕsij(1−ϕ)(tij−sij).
 MathType@MTEF@5@5@+=feaagaart1ev2aaatCvAUfKttLearuWrP9MDH5MBPbIqV92AaeXatLxBI9gBaebbnrfifHhDYfgasaacPC6xNi=xI8qiVKYPFjYdHaVhbbf9v8qqaqFr0xc9vqFj0dXdbba91qpepeI8k8fiI+fsY=rqGqVepae9pg0db9vqaiVgFr0xfr=xfr=xc9adbaqaaeGacaGaaiaabeqaaeqabiWaaaGcbaqbaeWabiGaaaqaaGqabiab=bfaqjabcUfaBjabcUha7jabcIcaOiabdsha0naaBaaaleaacqWGPbqAcqWGQbGAaeqaaOGaeiilaWIaem4Cam3aaSbaaSqaaiabdMgaPjabdQgaQbqabaGccqGGPaqkcqGG9bqFcqGG8baFiiGacqGF9oGBcqGGSaalcqGFvpGzcqGGSaalcqWGrbqucqGGDbqxaeaacqGH9aqpdaqeqbqaaiab=bfaqjabcUfaBjabdofatnaaBaaaleaacqWGPbqAcqWGQbGAaeqaaOGaeiiFaWNae4xVd4MaeiilaWIae4x1dyMaeiyxa01aaWbaaSqabeaacqWGZbWCdaWgaaadbaGaemyAaKMaemOAaOgabeaaaaGccqWFqbaucqGGBbWwcqWGgbGrdaWgaaWcbaGaemyAaKMaemOAaOgabeaakiabcYha8jab+17aUjabcYcaSiab+v9aMjabc2faDnaaCaaaleqabaGaemiDaq3aaSbaaWqaaiabdMgaPjabdQgaQbqabaWccqGHsislcqWGZbWCdaWgaaadbaGaemyAaKMaemOAaOgabeaaaaaaleaacqGGOaakcqWGPbqAcqGGSaalcqWGQbGAcqGGPaqkaeqaniabg+GivdaakeaaaeaacqGH9aqpdaqeqbqaaiabcIcaOiabigdaXiabgkHiTiab+17aUjabcMcaPmaaCaaaleqabaGaem4Cam3aaSbaaWqaaiabdMgaPjabdQgaQbqabaaaaOGae4xVd42aaWbaaSqabeaacqGGOaakcqWG0baDdaWgaaadbaGaemyAaKMaemOAaOgabeaaliabgkHiTiabdohaZnaaBaaameaacqWGPbqAcqWGQbGAaeqaaSGaeiykaKcaaaqaaiabcIcaOiabdMgaPjabcYcaSiabdQgaQjabcMcaPiabcQda6iabdgfarnaaBaaameaacqWGPbqAaeqaaSGaeyypa0Jaemyuae1aaSbaaWqaaiabdQgaQbqabaaaleqaniabg+GivdGcdaqeqbqaaiab+v9aMnaaCaaaleqabaGaem4Cam3aaSbaaWqaaiabdMgaPjabdQgaQbqabaaaaOGaeiikaGIaeGymaeJaeyOeI0Iae4x1dyMaeiykaKYaaWbaaSqabeaacqGGOaakcqWG0baDdaWgaaadbaGaemyAaKMaemOAaOgabeaaliabgkHiTiabdohaZnaaBaaameaacqWGPbqAcqWGQbGAaeqaaSGaeiykaKcaaaqaaiabcIcaOiabdMgaPjabcYcaSiabdQgaQjabcMcaPiabcQda6iabdgfarnaaBaaameaacqWGPbqAaeqaaSGaeyiyIKRaemyuae1aaSbaaWqaaiabdQgaQbqabaaaleqaniabg+GivdGccqGGUaGlaaaaaa@C688@

If we consider *Q*_*i *_to be a random variable for the cluster assignment of protein *i*, the entire cluster assignment is a Markov Random Field because (1) **P**[*Q*_*i *_= *k*] *> *0 and (2) its conditional distribution satisfies the Markov property,

**P**[*Q*_*i*_|*Q*_1_, ..., *Q*_*i*-1_, *Q*_*i*+1_, ..., *Q*_*N*_] = **P**[*Q*_*i*_|*Q*_*j*_, *j *∈ *Neighbor*(*i*)].

In other words, the joint probability **P**[*Q*] and the likelihood function only depend on the values of pairs of random variables *Q*_*i *_and *Q*_*j*_. In the terminology of Markov Random Fields as a statistical model [10,11,29], each protein *i *is a site that is labeled with its cluster *Q*_*i*_. The neighborhood of each site *i *consists of all those proteins *j *for which we have any observation for the protein pair (*i*, *j*), either interaction or non-interaction. To compute the cluster assignment *Q *using a Markov Random Field, we must define the potential function *U*(*Q*) which in this setting is derived from the negative logarithm of the likelihood.

The negative logarithm Λ of the above likelihood is,

Λ=∑(i,j):Qi=Qj[sij(−ln⁡(1−ν))+(tij−sij)(−ln⁡(ν))]+∑(i,j):Qi≠Qj[sij(−ln⁡(ϕ))+(tij−sij)(−ln⁡(1−ϕ))]
 MathType@MTEF@5@5@+=feaagaart1ev2aaatCvAUfKttLearuWrP9MDH5MBPbIqV92AaeXatLxBI9gBaebbnrfifHhDYfgasaacPC6xNi=xI8qiVKYPFjYdHaVhbbf9v8qqaqFr0xc9vqFj0dXdbba91qpepeI8k8fiI+fsY=rqGqVepae9pg0db9vqaiVgFr0xfr=xfr=xc9adbaqaaeGacaGaaiaabeqaaeqabiWaaaGcbaqbaeaabiGaaaqaaiabfU5amjabg2da9aqaamaaqafabaGaei4waSLaem4Cam3aaSbaaSqaaiabdMgaPjabdQgaQbqabaGccqGGOaakcqGHsislcyGGSbaBcqGGUbGBcqGGOaakcqaIXaqmcqGHsisliiGacqWF9oGBcqGGPaqkcqGGPaqkcqGHRaWkcqGGOaakcqWG0baDdaWgaaWcbaGaemyAaKMaemOAaOgabeaakiabgkHiTiabdohaZnaaBaaaleaacqWGPbqAcqWGQbGAaeqaaOGaeiykaKIaeiikaGIaeyOeI0IagiiBaWMaeiOBa4MaeiikaGIae8xVd4MaeiykaKIaeiykaKIaeiyxa0faleaacqGGOaakcqWGPbqAcqGGSaalcqWGQbGAcqGGPaqkcqGG6aGocqWGrbqudaWgaaadbaGaemyAaKgabeaaliabg2da9iabdgfarnaaBaaameaacqWGQbGAaeqaaaWcbeqdcqGHris5aaGcbaaabaGaey4kaSYaaabuaeaacqGGBbWwcqWGZbWCdaWgaaWcbaGaemyAaKMaemOAaOgabeaakiabcIcaOiabgkHiTiGbcYgaSjabc6gaUjabcIcaOiab=v9aMjabcMcaPiabcMcaPiabgUcaRiabcIcaOiabdsha0naaBaaaleaacqWGPbqAcqWGQbGAaeqaaOGaeyOeI0Iaem4Cam3aaSbaaSqaaiabdMgaPjabdQgaQbqabaGccqGGPaqkcqGGOaakcqGHsislcyGGSbaBcqGGUbGBcqGGOaakcqaIXaqmcqGHsislcqWFvpGzcqGGPaqkcqGGPaqkcqGGDbqxaSqaaiabcIcaOiabdMgaPjabcYcaSiabdQgaQjabcMcaPiabcQda6iabdgfarnaaBaaameaacqWGPbqAaeqaaSGaeyiyIKRaemyuae1aaSbaaWqaaiabdQgaQbqabaaaleqaniabggHiLdaaaaaa@9B74@

We then separate Λ into terms that depend on *Q *and terms that do not depend on *Q*. Λ can then be written as

Λ=∑(i,j):Qi≠Qjsijβ+(∑(i,j):Qi=Qj(tij−sij)α)+C,
 MathType@MTEF@5@5@+=feaagaart1ev2aaatCvAUfKttLearuWrP9MDH5MBPbIqV92AaeXatLxBI9gBaebbnrfifHhDYfgasaacPC6xNi=xI8qiVKYPFjYdHaVhbbf9v8qqaqFr0xc9vqFj0dXdbba91qpepeI8k8fiI+fsY=rqGqVepae9pg0db9vqaiVgFr0xfr=xfr=xc9adbaqaaeGacaGaaiaabeqaaeqabiWaaaGcbaGaeu4MdWKaeyypa0ZaaabuaeaacqWGZbWCdaWgaaWcbaGaemyAaKMaemOAaOgabeaaiiGakiab=j7aIjabgUcaRiabcIcaOaWcbaGaeiikaGIaemyAaKMaeiilaWIaemOAaOMaeiykaKIaeiOoaOJaemyuae1aaSbaaWqaaiabdMgaPbqabaWccqGHGjsUcqWGrbqudaWgaaadbaGaemOAaOgabeaaaSqab0GaeyyeIuoakmaaqafabaGaeiikaGIaemiDaq3aaSbaaSqaaiabdMgaPjabdQgaQbqabaGccqGHsislcqWGZbWCdaWgaaWcbaGaemyAaKMaemOAaOgabeaakiabcMcaPiab=f7aHjabcMcaPiabgUcaRiabdoeadjabcYcaSaWcbaGaeiikaGIaemyAaKMaeiilaWIaemOAaOMaeiykaKIaeiOoaOJaemyuae1aaSbaaWqaaiabdMgaPbqabaWccqGH9aqpcqWGrbqudaWgaaadbaGaemOAaOgabeaaaSqab0GaeyyeIuoaaaa@65A0@

where *α *= -ln(*ν*) + ln(1 - *φ*), and *β *= -ln(*φ*) + ln(1 - *ν*), and

C=∑(i,j)[(−sijln⁡(1−ν))+(−(tij−sij)ln⁡(1−ϕ))].
 MathType@MTEF@5@5@+=feaagaart1ev2aaatCvAUfKttLearuWrP9MDH5MBPbIqV92AaeXatLxBI9gBaebbnrfifHhDYfgasaacPC6xNi=xI8qiVKYPFjYdHaVhbbf9v8qqaqFr0xc9vqFj0dXdbba91qpepeI8k8fiI+fsY=rqGqVepae9pg0db9vqaiVgFr0xfr=xfr=xc9adbaqaaeGacaGaaiaabeqaaeqabiWaaaGcbaGaem4qamKaeyypa0ZaaabuaeaacqGGBbWwcqGGOaakcqGHsislcqWGZbWCdaWgaaWcbaGaemyAaKMaemOAaOgabeaakiGbcYgaSjabc6gaUjabcIcaOiabigdaXiabgkHiTGGaciab=17aUjabcMcaPiabcMcaPiabgUcaRiabcIcaOiabgkHiTiabcIcaOiabdsha0naaBaaaleaacqWGPbqAcqWGQbGAaeqaaOGaeyOeI0Iaem4Cam3aaSbaaSqaaiabdMgaPjabdQgaQbqabaGccqGGPaqkcyGGSbaBcqGGUbGBcqGGOaakcqaIXaqmcqGHsislcqWFvpGzcqGGPaqkcqGGPaqkcqGGDbqxcqGGUaGlaSqaaiabcIcaOiabdMgaPjabcYcaSiabdQgaQjabcMcaPaqab0GaeyyeIuoaaaa@5F0B@

*C *does not depend on *Q *and is thus irrelevant for minimization with respect to *Q*. The minimum is also unaffected by changes which leave *α *and *β *as long as the *ratio *of *α *and *β *unchanged. Incorporating these observations leads to the potential function

U(Q)=∑(i,j):Qi=Qj(tij−sij)+∑(i,j):Qi=Qjψsij,
 MathType@MTEF@5@5@+=feaagaart1ev2aaatCvAUfKttLearuWrP9MDH5MBPbIqV92AaeXatLxBI9gBaebbnrfifHhDYfgasaacPC6xNi=xI8qiVKYPFjYdHaVhbbf9v8qqaqFr0xc9vqFj0dXdbba91qpepeI8k8fiI+fsY=rqGqVepae9pg0db9vqaiVgFr0xfr=xfr=xc9adbaqaaeGacaGaaiaabeqaaeqabiWaaaGcbaGaemyvauLaeiikaGIaemyuaeLaeiykaKIaeyypa0ZaaabuaeaacqGGOaakcqWG0baDdaWgaaWcbaGaemyAaKMaemOAaOgabeaakiabgkHiTiabdohaZnaaBaaaleaacqWGPbqAcqWGQbGAaeqaaOGaeiykaKcaleaacqGGOaakcqWGPbqAcqGGSaalcqWGQbGAcqGGPaqkcqGG6aGocqWGrbqudaWgaaadbaGaemyAaKgabeaaliabg2da9iabdgfarnaaBaaameaacqWGQbGAaeqaaaWcbeqdcqGHris5aOGaey4kaSYaaabuaeaaiiGacqWFipqEcqWGZbWCdaWgaaWcbaGaemyAaKMaemOAaOgabeaaaeaacqGGOaakcqWGPbqAcqGGSaalcqWGQbGAcqGGPaqkcqGG6aGocqWGrbqudaWgaaadbaGaemyAaKgabeaaliabg2da9iabdgfarnaaBaaameaacqWGQbGAaeqaaaWcbeqdcqGHris5aOGaeiilaWcaaa@625F@

where ψ=−ln⁡(ϕ)+ln⁡(1−ν)−ln⁡(ν)+ln⁡(1−ϕ)=βα
 MathType@MTEF@5@5@+=feaagaart1ev2aaatCvAUfKttLearuWrP9MDH5MBPbIqV92AaeXatLxBI9gBaebbnrfifHhDYfgasaacPC6xNi=xH8viVGI8Gi=hEeeu0xXdbba9frFj0xb9qqpG0dXdb9aspeI8k8fiI+fsY=rqGqVepae9pg0db9vqaiVgFr0xfr=xfr=xc9adbaqaaeGacaGaaiaabeqaaeqabiWaaaGcbaacciGae8hYdKNaeyypa0tcfa4aaSaaaeaacqGHsislcyGGSbaBcqGGUbGBcqGGOaakcqWFvpGzcqGGPaqkcqGHRaWkcyGGSbaBcqGGUbGBcqGGOaakcqaIXaqmcqGHsislcqWF9oGBcqGGPaqkaeaacqGHsislcyGGSbaBcqGGUbGBcqGGOaakcqWF9oGBcqGGPaqkcqGHRaWkcyGGSbaBcqGGUbGBcqGGOaakcqaIXaqmcqGHsislcqWFvpGzcqGGPaqkaaGccqGH9aqpjuaGdaWcaaqaaiab=j7aIbqaaiab=f7aHbaaaaa@5454@. It is noteworthy that this potential function is the same for pairs of *φ *and *ν *that are related by a common *ψ*. Minimization with respect to *Q*_*i*_, *ν *and *φ *yields our desired solution.

### Mean field annealing: a solution technique for Markov Random Fields

Mean field annealing is a popular technique to compute a maximum-likelihood label assignment for Markov random fields [10,29,30]. We will replace the random variables *Q*_*i *_with probabilities

*q*_*ik *_= **P**[*Q*_*i *_= *k*].

It is well known (e.g., see [29]) that the joint probability distribution of *Q *is a Gibbs distribution, given by

**P**[*Q*] = *Z*^-1 ^exp[-*γU*(*Q*)],

where *U*(*Q*) is the potential function (Eq. 4) and *γ *is the annealing factor. *Z *is the normalization factor, also called the partition function, with

Z=∑Qexp⁡[−γU(Q)].
 MathType@MTEF@5@5@+=feaagaart1ev2aaatCvAUfKttLearuWrP9MDH5MBPbIqV92AaeXatLxBI9gBaebbnrfifHhDYfgasaacPC6xNi=xI8qiVKYPFjYdHaVhbbf9v8qqaqFr0xc9vqFj0dXdbba91qpepeI8k8fiI+fsY=rqGqVepae9pg0db9vqaiVgFr0xfr=xfr=xc9adbaqaaeGacaGaaiaabeqaaeqabiWaaaGcbaGaemOwaOLaeyypa0ZaaabuaeaacyGGLbqzcqGG4baEcqGGWbaCcqGGBbWwcqGHsisliiGacqWFZoWzcqWGvbqvcqGGOaakcqWGrbqucqGGPaqkcqGGDbqxcqGGUaGlaSqaaiabdgfarbqab0GaeyyeIuoaaaa@3FF5@

Mean field theory provides a framework to compute *q*_*ik*_. For our clustering problem, we will apply it to estimate the probability of assigning protein *i *to a cluster *k*, call q^ik
 MathType@MTEF@5@5@+=feaagaart1ev2aaatCvAUfKttLearuWrP9MDH5MBPbIqV92AaeXatLxBI9gBaebbnrfifHhDYfgasaacPC6xNi=xH8viVGI8Gi=hEeeu0xXdbba9frFj0xb9qqpG0dXdb9aspeI8k8fiI+fsY=rqGqVepae9pg0db9vqaiVgFr0xfr=xfr=xc9adbaqaaeGacaGaaiaabeqaaeqabiWaaaGcbaGafmyCaeNbaKaadaWgaaWcbaGaemyAaKMaem4AaSgabeaaaaa@3034@, defined by

q^ik=P[Qi=k|Qj,j≠i]∑l=1KP[Qi=l|Qj,j≠i]=exp⁡[−γU(Qi=k|Qj,j≠i]∑l=1Kexp⁡[−γU[Qi=l|Qj,j≠i)].
 MathType@MTEF@5@5@+=feaagaart1ev2aaatCvAUfKttLearuWrP9MDH5MBPbIqV92AaeXatLxBI9gBaebbnrfifHhDYfgasaacPC6xNi=xI8qiVKYPFjYdHaVhbbf9v8qqaqFr0xc9vqFj0dXdbba91qpepeI8k8fiI+fsY=rqGqVepae9pg0db9vqaiVgFr0xfr=xfr=xc9adbaqaaeGacaGaaiaabeqaaeqabiWaaaGcbaqbaeaabiWaaaqaaiqbdghaXzaajaWaaSbaaSqaaiabdMgaPjabdUgaRbqabaaakeaacqGH9aqpaKqbagaadaWcaaqaaGqabiab=bfaqjabcUfaBjabdgfarnaaBaaabaGaemyAaKgabeaacqGH9aqpcqWGRbWAcqGG8baFcqWGrbqudaWgaaqaaiabdQgaQbqabaGaeiilaWIaemOAaOMaeyiyIKRaemyAaKMaeiyxa0fabaWaaabCaeaacqWFqbaucqGGBbWwcqWGrbqudaWgaaqaaiabdMgaPbqabaGaeyypa0JaemiBaWMaeiiFaWNaemyuae1aaSbaaeaacqWGQbGAaeqaaiabcYcaSiabdQgaQjabgcMi5kabdMgaPjabc2faDbqaaiabdYgaSjabg2da9iabigdaXaqaaiabdUealbGaeyyeIuoaaaaakeaaaeaacqGH9aqpaeaajuaGdaWcaaqaaiGbcwgaLjabcIha4jabcchaWjabcUfaBjabgkHiTGGaciab+n7aNjabdwfavjabcIcaOiabdgfarnaaBaaabaGaemyAaKgabeaacqGH9aqpcqWGRbWAcqGG8baFcqWGrbqudaWgaaqaaiabdQgaQbqabaGaeiilaWIaemOAaOMaeyiyIKRaemyAaKMaeiyxa0fabaWaaabCaeaacyGGLbqzcqGG4baEcqGGWbaCcqGGBbWwcqGHsislcqGFZoWzcqWGvbqvcqGGBbWwcqWGrbqudaWgaaqaaiabdMgaPbqabaGaeyypa0JaemiBaWMaeiiFaWNaemyuae1aaSbaaeaacqWGQbGAaeqaaiabcYcaSiabdQgaQjabgcMi5kabdMgaPjabcMcaPiabc2faDbqaaiabdYgaSjabg2da9iabigdaXaqaaiabdUealbGaeyyeIuoaaaGccqGGUaGlaaaaaa@9BAC@

Computing the actual conditional energy function is not feasible because it requires us to evaluate the clustering assignment of the whole MRF, which is not known. By assuming the Markov property and replacing the random variables *Q*_*i *_and *Q*_*j *_with the expected values of cluster assignments within each protein's neighborhood (the mean field), we can estimate *U*(*Q*_*i *_= *k*|*Q*_*j*_, *j *≠ *i*) by

U(Qi=k|Qj,j≠i)=U(Qi=k|Qj,j∈Neighbor(i))=∑j∈Neighbor(i)(∑l=1Kqilqjl)(tij−sij)+(1−∑l=1Kqilqjl)ψsij.
 MathType@MTEF@5@5@+=feaagaart1ev2aaatCvAUfKttLearuWrP9MDH5MBPbIqV92AaeXatLxBI9gBaebbnrfifHhDYfgasaacPC6xNi=xI8qiVKYPFjYdHaVhbbf9v8qqaqFr0xc9vqFj0dXdbba91qpepeI8k8fiI+fsY=rqGqVepae9pg0db9vqaiVgFr0xfr=xfr=xc9adbaqaaeGacaGaaiaabeqaaeqabiWaaaGcbaqbaeaabiWaaaqaaiabdwfavjabcIcaOiabdgfarnaaBaaaleaacqWGPbqAaeqaaOGaeyypa0Jaem4AaSMaeiiFaWNaemyuae1aaSbaaSqaaiabdQgaQbqabaGccqGGSaalcqWGQbGAcqGHGjsUcqWGPbqAcqGGPaqkaeaacqGH9aqpaeaacqWGvbqvcqGGOaakcqWGrbqudaWgaaWcbaGaemyAaKgabeaakiabg2da9iabdUgaRjabcYha8jabdgfarnaaBaaaleaacqWGQbGAaeqaaOGaeiilaWIaemOAaOMaeyicI4SaemOta4KaemyzauMaemyAaKMaem4zaCMaemiAaGMaemOyaiMaem4Ba8MaemOCaiNaeiikaGIaemyAaKMaeiykaKIaeiykaKcabaaabaGaeyypa0dabaWaaabuaeaacqGGOaakdaaeWbqaaiabdghaXnaaBaaaleaacqWGPbqAcqWGSbaBaeqaaOGaemyCae3aaSbaaSqaaiabdQgaQjabdYgaSbqabaGccqGGPaqkcqGGOaakcqWG0baDdaWgaaWcbaGaemyAaKMaemOAaOgabeaakiabgkHiTiabdohaZnaaBaaaleaacqWGPbqAcqWGQbGAaeqaaOGaeiykaKIaey4kaSIaeiikaGIaeGymaeJaeyOeI0YaaabCaeaacqWGXbqCdaWgaaWcbaGaemyAaKMaemiBaWgabeaakiabdghaXnaaBaaaleaacqWGQbGAcqWGSbaBaeqaaaqaaiabdYgaSjabg2da9iabigdaXaqaaiabdUealbqdcqGHris5aOGaeiykaKccciGae8hYdKNaem4Cam3aaSbaaSqaaiabdMgaPjabdQgaQbqabaaabaGaemiBaWMaeyypa0JaeGymaedabaGaem4saSeaniabggHiLdGccqGGUaGlaSqaaiabdQgaQjabgIGiolabd6eaojabdwgaLjabdMgaPjabdEgaNjabdIgaOjabdkgaIjabd+gaVjabdkhaYjabcIcaOiabdMgaPjabcMcaPaqab0GaeyyeIuoaaaaaaa@A7B3@

We evaluate the conditional energy function only at a fixed point by assuming that *q*_*ik *_= 1 and *q*_*il *_= 0 for *l *≠ *k*. We can then approximate *U*(*Q*_*i *_= *k*|*Q*_*j*_, *j *≠ *i*) by

Cik=∑j∈Neighbor(i)qjk(tij−sij)+(1−qik)ψsij.
 MathType@MTEF@5@5@+=feaagaart1ev2aaatCvAUfKttLearuWrP9MDH5MBPbIqV92AaeXatLxBI9gBaebbnrfifHhDYfgasaacPC6xNi=xI8qiVKYPFjYdHaVhbbf9v8qqaqFr0xc9vqFj0dXdbba91qpepeI8k8fiI+fsY=rqGqVepae9pg0db9vqaiVgFr0xfr=xfr=xc9adbaqaaeGacaGaaiaabeqaaeqabiWaaaGcbaGaem4qam0aaSbaaSqaaiabdMgaPjabdUgaRbqabaGccqGH9aqpdaaeqbqaaiabdghaXnaaBaaaleaacqWGQbGAcqWGRbWAaeqaaOGaeiikaGIaemiDaq3aaSbaaSqaaiabdMgaPjabdQgaQbqabaaabaGaemOAaOMaeyicI4SaemOta4KaemyzauMaemyAaKMaem4zaCMaemiAaGMaemOyaiMaem4Ba8MaemOCaiNaeiikaGIaemyAaKMaeiykaKcabeqdcqGHris5aOGaeyOeI0Iaem4Cam3aaSbaaSqaaiabdMgaPjabdQgaQbqabaGccqGGPaqkcqGHRaWkcqGGOaakcqaIXaqmcqGHsislcqWGXbqCdaWgaaWcbaGaemyAaKMaem4AaSgabeaakiabcMcaPGGaciab=H8a5jabdohaZnaaBaaaleaacqWGPbqAcqWGQbGAaeqaaOGaeiOla4caaa@636A@

Thus, the assignment probability *q*_*ik *_can be computed by

q^ik=exp⁡[−γCik]∑l=1Kexp⁡[−γCil].
 MathType@MTEF@5@5@+=feaagaart1ev2aaatCvAUfKttLearuWrP9MDH5MBPbIqV92AaeXatLxBI9gBaebbnrfifHhDYfgasaacPC6xNi=xI8qiVKYPFjYdHaVhbbf9v8qqaqFr0xc9vqFj0dXdbba91qpepeI8k8fiI+fsY=rqGqVepae9pg0db9vqaiVgFr0xfr=xfr=xc9adbaqaaeGacaGaaiaabeqaaeqabiWaaaGcbaGafmyCaeNbaKaadaWgaaWcbaGaemyAaKMaem4AaSgabeaakiabg2da9KqbaoaalaaabaGagiyzauMaeiiEaGNaeiiCaaNaei4waSLaeyOeI0ccciGae83SdCMaem4qam0aaSbaaeaacqWGPbqAcqWGRbWAaeqaaiabc2faDbqaamaaqahabaGagiyzauMaeiiEaGNaeiiCaaNaei4waSLaeyOeI0Iae83SdCMaem4qam0aaSbaaeaacqWGPbqAcqWGSbaBaeqaaiabc2faDbqaaiabdYgaSjabg2da9iabigdaXaqaaiabdUealbGaeyyeIuoaaaGccqGGUaGlaaa@5426@

In terms of computation, notice that in order to find the mean field at *i*, we needs to know the mean field at the neighbors of *i*. The mean field is usually computed by iterative procedures, details of our approach are shown in Table 7. The annealing procedure will drive *q *to discrete distribution. For *γ *→ ∞, *q*_*ik *_→ 1 for some *k *= *l *and *q*_*ik *_→ 0 for some *k *≠ *l*. As a result, the membership probability *q *becomes a discrete cluster assignment.

**Table 7:** 

Algorithm: Mean field annealing
\SetKwInOut{Input}{Input}
\SetKwInOut{Output}{Output}
\Input{A set of observations (*t*_*ij*_, *s*_*ij*_) for each pair (*i*, *j*), *ψ*, a number of clusters *K*}
\Output{A probability *q*_*ik *_for a node *i *belonging to a cluster *k *for all *i *and for all *k*}
Initialize *q *to random values;
Initialize annealing factor *γ*;
**While***γ *<*γ*_max _**Repeat***q *converges
**ForAll***i *∈ *V*
**ForAll***k *∈ *K*
Cik=∑j∈Neighbor(i)qjk(tij−sij)+(1−qjk)ψsij MathType@MTEF@5@5@+=feaagaart1ev2aaatCvAUfKttLearuWrP9MDH5MBPbIqV92AaeXatLxBI9gBaebbnrfifHhDYfgasaacPC6xNi=xH8viVGI8Gi=hEeeu0xXdbba9frFj0xb9qqpG0dXdb9aspeI8k8fiI+fsY=rqGqVepae9pg0db9vqaiVgFr0xfr=xfr=xc9adbaqaaeGacaGaaiaabeqaaeqabiWaaaGcbaGaem4qam0aaSbaaSqaaiabdMgaPjabdUgaRbqabaGccqGH9aqpdaaeqbqaaiabdghaXnaaBaaaleaacqWGQbGAcqWGRbWAaeqaaOGaeiikaGIaemiDaq3aaSbaaSqaaiabdMgaPjabdQgaQbqabaGccqGHsislcqWGZbWCdaWgaaWcbaGaemyAaKMaemOAaOgabeaakiabcMcaPiabgUcaRiabcIcaOiabigdaXiabgkHiTiabdghaXnaaBaaaleaacqWGQbGAcqWGRbWAaeqaaOGaeiykaKccciGae8hYdKNaem4Cam3aaSbaaSqaaiabdMgaPjabdQgaQbqabaaabaGaemOAaOMaeyicI4SaemOta4KaemyzauMaemyAaKMaem4zaCMaemiAaGMaemOyaiMaem4Ba8MaemOCaiNaeiikaGIaemyAaKMaeiykaKcabeqdcqGHris5aaaa@6230@
**ForAll***k *∈ *K*
q^ik=exp⁡(−γCik)∑l=1Kexp⁡(−γCil) MathType@MTEF@5@5@+=feaagaart1ev2aaatCvAUfKttLearuWrP9MDH5MBPbIqV92AaeXatLxBI9gBaebbnrfifHhDYfgasaacPC6xNi=xH8viVGI8Gi=hEeeu0xXdbba9frFj0xb9qqpG0dXdb9aspeI8k8fiI+fsY=rqGqVepae9pg0db9vqaiVgFr0xfr=xfr=xc9adbaqaaeGacaGaaiaabeqaaeqabiWaaaGcbaGafmyCaeNbaKaadaWgaaWcbaGaemyAaKMaem4AaSgabeaakiabg2da9KqbaoaalaaabaGagiyzauMaeiiEaGNaeiiCaaNaeiikaGIaeyOeI0ccciGae83SdCMaem4qam0aaSbaaeaacqWGPbqAcqWGRbWAaeqaaiabcMcaPaqaamaaqahabaGagiyzauMaeiiEaGNaeiiCaaNaeiikaGIaeyOeI0Iae83SdCMaem4qam0aaSbaaeaacqWGPbqAcqWGSbaBaeqaaiabcMcaPaqaaiabdYgaSjabg2da9iabigdaXaqaaiabdUealbGaeyyeIuoaaaaaaa@514E@
**ForAll***k *∈ *K*
*q*_*ik *_= q^ik MathType@MTEF@5@5@+=feaagaart1ev2aaatCvAUfKttLearuWrP9MDH5MBPbIqV92AaeXatLxBI9gBaebbnrfifHhDYfgasaacPC6xNi=xH8viVGI8Gi=hEeeu0xXdbba9frFj0xb9qqpG0dXdb9aspeI8k8fiI+fsY=rqGqVepae9pg0db9vqaiVgFr0xfr=xfr=xc9adbaqaaeGacaGaaiaabeqaaeqabiWaaaGcbaGafmyCaeNbaKaadaWgaaWcbaGaemyAaKMaem4AaSgabeaaaaa@3034@
Increase *γ*;

#### Estimation of false negative and false positive rate

Given a cluster assignment *Q*, we can estimate the error rate *ν *and *φ *by minimizing equation Eq. 2 with respect to *ν *and *φ*. The derivative of Eq. 2 with respect to *ν *is

∂Λ∂ν=a1−ν−bν,
 MathType@MTEF@5@5@+=feaagaart1ev2aaatCvAUfKttLearuWrP9MDH5MBPbIqV92AaeXatLxBI9gBaebbnrfifHhDYfgasaacPC6xNi=xI8qiVKYPFjYdHaVhbbf9v8qqaqFr0xc9vqFj0dXdbba91qpepeI8k8fiI+fsY=rqGqVepae9pg0db9vqaiVgFr0xfr=xfr=xc9adbaqaaeGacaGaaiaabeqaaeqabiWaaaGcbaqcfa4aaSaaaeaacqGHciITcqqHBoataeaacqGHciITiiGacqWF9oGBaaGccqGH9aqpjuaGdaWcaaqaaiabdggaHbqaaiabigdaXiabgkHiTiab=17aUbaakiabgkHiTKqbaoaalaaabaGaemOyaigabaGae8xVd4gaaOGaeiilaWcaaa@3EC7@

where a=∑(i,j):Qi=Qjsij
 MathType@MTEF@5@5@+=feaagaart1ev2aaatCvAUfKttLearuWrP9MDH5MBPbIqV92AaeXatLxBI9gBaebbnrfifHhDYfgasaacPC6xNi=xH8viVGI8Gi=hEeeu0xXdbba9frFj0xb9qqpG0dXdb9aspeI8k8fiI+fsY=rqGqVepae9pg0db9vqaiVgFr0xfr=xfr=xc9adbaqaaeGacaGaaiaabeqaaeqabiWaaaGcbaGaemyyaeMaeyypa0ZaaabuaeaacqWGZbWCdaWgaaWcbaGaemyAaKMaemOAaOgabeaaaeaacqGGOaakcqWGPbqAcqGGSaalcqWGQbGAcqGGPaqkcqGG6aGocqWGrbqudaWgaaadbaGaemyAaKgabeaaliabg2da9iabdgfarnaaBaaameaacqWGQbGAaeqaaaWcbeqdcqGHris5aaaa@4159@, and b=∑(i,j):Qi=Qj(tij−sij)
 MathType@MTEF@5@5@+=feaagaart1ev2aaatCvAUfKttLearuWrP9MDH5MBPbIqV92AaeXatLxBI9gBaebbnrfifHhDYfgasaacPC6xNi=xH8viVGI8Gi=hEeeu0xXdbba9frFj0xb9qqpG0dXdb9aspeI8k8fiI+fsY=rqGqVepae9pg0db9vqaiVgFr0xfr=xfr=xc9adbaqaaeGacaGaaiaabeqaaeqabiWaaaGcbaGaemOyaiMaeyypa0ZaaabuaeaacqGGOaakcqWG0baDdaWgaaWcbaGaemyAaKMaemOAaOgabeaakiabgkHiTiabdohaZnaaBaaaleaacqWGPbqAcqWGQbGAaeqaaOGaeiykaKcaleaacqGGOaakcqWGPbqAcqGGSaalcqWGQbGAcqGGPaqkcqGG6aGocqWGrbqudaWgaaadbaGaemyAaKgabeaaliabg2da9iabdgfarnaaBaaameaacqWGQbGAaeqaaaWcbeqdcqGHris5aaaa@486E@. The derivative of Eq. 2 with respect to *φ *is

∂Λ∂ϕ=−cϕ+d1−ϕ,
 MathType@MTEF@5@5@+=feaagaart1ev2aaatCvAUfKttLearuWrP9MDH5MBPbIqV92AaeXatLxBI9gBaebbnrfifHhDYfgasaacPC6xNi=xI8qiVKYPFjYdHaVhbbf9v8qqaqFr0xc9vqFj0dXdbba91qpepeI8k8fiI+fsY=rqGqVepae9pg0db9vqaiVgFr0xfr=xfr=xc9adbaqaaeGacaGaaiaabeqaaeqabiWaaaGcbaqcfa4aaSaaaeaacqGHciITcqqHBoataeaacqGHciITiiGacqWFvpGzaaGccqGH9aqpcqGHsisljuaGdaWcaaqaaiabdogaJbqaaiab=v9aMbaakiabgUcaRKqbaoaalaaabaGaemizaqgabaGaeGymaeJaeyOeI0Iae8x1dygaaOGaeiilaWcaaa@3FE1@

where c=∑(i,j):Qi≠Qjsij
 MathType@MTEF@5@5@+=feaagaart1ev2aaatCvAUfKttLearuWrP9MDH5MBPbIqV92AaeXatLxBI9gBaebbnrfifHhDYfgasaacPC6xNi=xH8viVGI8Gi=hEeeu0xXdbba9frFj0xb9qqpG0dXdb9aspeI8k8fiI+fsY=rqGqVepae9pg0db9vqaiVgFr0xfr=xfr=xc9adbaqaaeGacaGaaiaabeqaaeqabiWaaaGcbaGaem4yamMaeyypa0ZaaabuaeaacqWGZbWCdaWgaaWcbaGaemyAaKMaemOAaOgabeaaaeaacqGGOaakcqWGPbqAcqGGSaalcqWGQbGAcqGGPaqkcqGG6aGocqWGrbqudaWgaaadbaGaemyAaKgabeaaliabgcMi5kabdgfarnaaBaaameaacqWGQbGAaeqaaaWcbeqdcqGHris5aaaa@421E@, and d=∑(i,j):Qi≠Qj(tij−sij)
 MathType@MTEF@5@5@+=feaagaart1ev2aaatCvAUfKttLearuWrP9MDH5MBPbIqV92AaeXatLxBI9gBaebbnrfifHhDYfgasaacPC6xNi=xH8viVGI8Gi=hEeeu0xXdbba9frFj0xb9qqpG0dXdb9aspeI8k8fiI+fsY=rqGqVepae9pg0db9vqaiVgFr0xfr=xfr=xc9adbaqaaeGacaGaaiaabeqaaeqabiWaaaGcbaGaemizaqMaeyypa0ZaaabuaeaacqGGOaakcqWG0baDdaWgaaWcbaGaemyAaKMaemOAaOgabeaakiabgkHiTiabdohaZnaaBaaaleaacqWGPbqAcqWGQbGAaeqaaOGaeiykaKcaleaacqGGOaakcqWGPbqAcqGGSaalcqWGQbGAcqGGPaqkcqGG6aGocqWGrbqudaWgaaadbaGaemyAaKgabeaaliabgcMi5kabdgfarnaaBaaameaacqWGQbGAaeqaaaWcbeqdcqGHris5aaaa@4933@. Setting Eq. 8 and Eq. 9 to zero, the solutions for optimal error rates ν* and φ*, given the cluster assignments *Q*, are

ν∗=∑(i,j):Qi=Qj(tij−sij)∑(i,j):Qi=Qjtij,
 MathType@MTEF@5@5@+=feaagaart1ev2aaatCvAUfKttLearuWrP9MDH5MBPbIqV92AaeXatLxBI9gBaebbnrfifHhDYfgasaacPC6xNi=xI8qiVKYPFjYdHaVhbbf9v8qqaqFr0xc9vqFj0dXdbba91qpepeI8k8fiI+fsY=rqGqVepae9pg0db9vqaiVgFr0xfr=xfr=xc9adbaqaaeGacaGaaiaabeqaaeqabiWaaaGcbaacciGae8xVd42aaWbaaSqabeaacqGHxiIkaaGccqGH9aqpjuaGdaWcaaqaamaaqafabaGaeiikaGIaemiDaq3aaSbaaeaacqWGPbqAcqWGQbGAaeqaaiabgkHiTiabdohaZnaaBaaabaGaemyAaKMaemOAaOgabeaacqGGPaqkaeaacqGGOaakcqWGPbqAcqGGSaalcqWGQbGAcqGGPaqkcqGG6aGocqWGrbqudaWgaaqaaiabdMgaPbqabaGaeyypa0Jaemyuae1aaSbaaeaacqWGQbGAaeqaaaqabiabggHiLdaabaWaaabuaeaacqWG0baDdaWgaaqaaiabdMgaPjabdQgaQbqabaaabaGaeiikaGIaemyAaKMaeiilaWIaemOAaOMaeiykaKIaeiOoaOJaemyuae1aaSbaaeaacqWGPbqAaeqaaiabg2da9iabdgfarnaaBaaabaGaemOAaOgabeaaaeqacqGHris5aaaakiabcYcaSaaa@5E5D@

and

ϕ∗=∑(i,j):Qi≠Qjsij∑(i,j):Qi≠Qjtij.
 MathType@MTEF@5@5@+=feaagaart1ev2aaatCvAUfKttLearuWrP9MDH5MBPbIqV92AaeXatLxBI9gBaebbnrfifHhDYfgasaacPC6xNi=xI8qiVKYPFjYdHaVhbbf9v8qqaqFr0xc9vqFj0dXdbba91qpepeI8k8fiI+fsY=rqGqVepae9pg0db9vqaiVgFr0xfr=xfr=xc9adbaqaaeGacaGaaiaabeqaaeqabiWaaaGcbaacciGae8x1dy2aaWbaaSqabeaacqGHxiIkaaGccqGH9aqpjuaGdaWcaaqaamaaqafabaGaem4Cam3aaSbaaeaacqWGPbqAcqWGQbGAaeqaaaqaaiabcIcaOiabdMgaPjabcYcaSiabdQgaQjabcMcaPiabcQda6iabdgfarnaaBaaabaGaemyAaKgabeaacqGHGjsUcqWGrbqudaWgaaqaaiabdQgaQbqabaaabeGaeyyeIuoaaeaadaaeqbqaaiabdsha0naaBaaabaGaemyAaKMaemOAaOgabeaaaeaacqGGOaakcqWGPbqAcqGGSaalcqWGQbGAcqGGPaqkcqGG6aGocqWGrbqudaWgaaqaaiabdMgaPbqabaGaeyiyIKRaemyuae1aaSbaaeaacqWGQbGAaeqaaaqabiabggHiLdaaaOGaeiOla4caaa@590A@

When evaluating the likelihood of a particular solution *Q*, we use ν* and φ* that maximizes the likelihood.

#### Minimization strategy

Each run of Mean Field Annealing requires two inputs, the number of clusters *K *and the error rate ratio *ψ*. We find values for both inputs that maximize the likelihood of solution *Q *by repeatedly optimizing *Q *using Mean Field Annealing for different values of *K *and *ψ*. Our tests show that on a large scale, the likelihood is roughly convex with respect to these two values, but unfortunately with smaller scale local minima interspersed. To avoid getting stuck in these local minima, we perform iterative line minimization, alternating between minimizing with respect to *K *and *ψ*, while holding the other constant. At each step, we computed five to seven values within a progressively smaller range. In our tests, three iterations were sufficient for converging upon the maximum likelihood (minimum negative log-likelihood).

### Implementation

We implemented the Mean Field Annealing algorithm in C++. The running time of Mean Field Annealing is quadratic in the number of nodes, that is *O*(*K*|*V*|^2^). On a data set of about 3000 proteins, a single minimization for a fixed number of clusters takes an average of 10 hours of CPU time on Athlon 3 GHz processor.

### Accuracy

*Complex-coverage *(denoted CO) characterizes the average coverage of complexes by a clustering result,

CO=∑i=1nNi(max⁡jCOij)∑i=1nNi,
 MathType@MTEF@5@5@+=feaagaart1ev2aaatCvAUfKttLearuWrP9MDH5MBPbIqV92AaeXatLxBI9gBaebbnrfifHhDYfgasaacPC6xNi=xI8qiVKYPFjYdHaVhbbf9v8qqaqFr0xc9vqFj0dXdbba91qpepeI8k8fiI+fsY=rqGqVepae9pg0db9vqaiVgFr0xfr=xfr=xc9adbaqaaeGacaGaaiaabeqaaeqabiWaaaGcbaGaee4qamKaee4ta8Kaeyypa0tcfa4aaSaaaeaadaaeWbqaaiabd6eaonaaBaaabaGaemyAaKgabeaacqGGOaakdaWfqaqaaiGbc2gaTjabcggaHjabcIha4bqaaiabdQgaQbqabaGaee4qamKaee4ta80aaSbaaeaacqWGPbqAcqWGQbGAaeqaaiabcMcaPaqaaiabdMgaPjabg2da9iabigdaXaqaaiabd6gaUbGaeyyeIuoaaeaadaaeWbqaaiabd6eaonaaBaaabaGaemyAaKgabeaaaeaacqWGPbqAcqGH9aqpcqaIXaqmaeaacqWGUbGBaiabggHiLdaaaOGaeiilaWcaaa@5051@

where CO_*ij *_= *T*_*ij*_*/N*_*i*_, *N*_*i *_is the number of proteins in the complex *i*.

A *positive-predictive value *(denoted PPV) is the proportion of proteins in cluster *j *that belong to complex *i*, relative to the total number of members of this cluster assigned to all complexes.

PPVij=Tij∑i=1nTij=TijT.j.
 MathType@MTEF@5@5@+=feaagaart1ev2aaatCvAUfKttLearuWrP9MDH5MBPbIqV92AaeXatLxBI9gBaebbnrfifHhDYfgasaacPC6xNi=xI8qiVKYPFjYdHaVhbbf9v8qqaqFr0xc9vqFj0dXdbba91qpepeI8k8fiI+fsY=rqGqVepae9pg0db9vqaiVgFr0xfr=xfr=xc9adbaqaaeGacaGaaiaabeqaaeqabiWaaaGcbaGaeeiuaaLaeeiuaaLaeeOvay1aaSbaaSqaaiabdMgaPjabdQgaQbqabaGccqGH9aqpjuaGdaWcaaqaaiabdsfaunaaBaaabaGaemyAaKMaemOAaOgabeaaaeaadaaeWbqaaiabdsfaunaaBaaabaGaemyAaKMaemOAaOgabeaaaeaacqWGPbqAcqGH9aqpcqaIXaqmaeaacqWGUbGBaiabggHiLdaaaOGaeyypa0tcfa4aaSaaaeaacqWGubavdaWgaaqaaiabdMgaPjabdQgaQbqabaaabaGaemivaq1aaSbaaeaacqGGUaGlcqWGQbGAaeqaaaaakiabc6caUaaa@4D61@

Note that the normalization is not the size of cluster *j*, but the marginal sum of a column *j *which can be different from the cluster size because some proteins belong to more than one complex. To characterize the general positive-predictive value of a clustering result as a whole, we use the following weighted average quantity,

PPV=∑j=1mT.j(max⁡iPPVij)∑j=1mT.j.
 MathType@MTEF@5@5@+=feaagaart1ev2aaatCvAUfKttLearuWrP9MDH5MBPbIqV92AaeXatLxBI9gBaebbnrfifHhDYfgasaacPC6xNi=xI8qiVKYPFjYdHaVhbbf9v8qqaqFr0xc9vqFj0dXdbba91qpepeI8k8fiI+fsY=rqGqVepae9pg0db9vqaiVgFr0xfr=xfr=xc9adbaqaaeGacaGaaiaabeqaaeqabiWaaaGcbaGaeeiuaaLaeeiuaaLaeeOvayLaeyypa0tcfa4aaSaaaeaadaaeWbqaaiabdsfaunaaBaaabaGaeiOla4IaemOAaOgabeaacqGGOaakdaWfqaqaaiGbc2gaTjabcggaHjabcIha4bqaaiabdMgaPbqabaGaeeiuaaLaeeiuaaLaeeOvay1aaSbaaeaacqWGPbqAcqWGQbGAaeqaaiabcMcaPaqaaiabdQgaQjabg2da9iabigdaXaqaaiabd2gaTbGaeyyeIuoaaeaadaaeWbqaaiabdsfaunaaBaaabaGaeiOla4IaemOAaOgabeaaaeaacqWGQbGAcqGH9aqpcqaIXaqmaeaacqWGTbqBaiabggHiLdaaaiabc6caUaaa@54CB@

The accuracy *Acc *is a geometric average between complex-coverage and positive-predictive value, Acc=CO×PPV
 MathType@MTEF@5@5@+=feaagaart1ev2aaatCvAUfKttLearuWrP9MDH5MBPbIqV92AaeXatLxBI9gBaebbnrfifHhDYfgasaacPC6xNi=xH8viVGI8Gi=hEeeu0xXdbba9frFj0xb9qqpG0dXdb9aspeI8k8fiI+fsY=rqGqVepae9pg0db9vqaiVgFr0xfr=xfr=xc9adbaqaaeGacaGaaiaabeqaaeqabiWaaaGcbaGaemyqaeKaem4yamMaem4yamMaeyypa0ZaaOaaaeaacqqGdbWqcqqGpbWtcqGHxdaTcqqGqbaucqqGqbaucqqGwbGvaSqabaaaaa@3867@.

### All-pairs comparison: sensitivity and specificity

For the second procedure, we use the standard all-pairs sensitivity (SN) and specificity (SP). We refer to an (unordered) pair of proteins from the same complex as a *true *pair, and to a pair of proteins from the same cluster as a *predicted *pair. We call a true predicted pair *true positive *(TP), a true pair which has not been predicted *false negative *(FN), a false pair predicted to be from the same complex *false positive *(FP) and a correctly predicted false pair *true negative *(TN). The following quantities summarize the performance of all-pair comparison: *Sensitivity*, SN=#TP#TP+#FN
 MathType@MTEF@5@5@+=feaagaart1ev2aaatCvAUfKttLearuWrP9MDH5MBPbIqV92AaeXatLxBI9gBaebbnrfifHhDYfgasaacPC6xNi=xH8viVGI8Gi=hEeeu0xXdbba9frFj0xb9qqpG0dXdb9aspeI8k8fiI+fsY=rqGqVepae9pg0db9vqaiVgFr0xfr=xfr=xc9adbaqaaeGacaGaaiaabeqaaeqabiWaaaGcbaGaee4uamLaeeOta4Kaeyypa0tcfa4aaSaaaeaacqGGJaWicqWGubavcqWGqbauaeaacqGGJaWicqWGubavcqWGqbaucqGHRaWkcqGGJaWicqWGgbGrcqWGobGtaaaaaa@3A01@ and *Specificity*, SP=#TP#TP+#FP
 MathType@MTEF@5@5@+=feaagaart1ev2aaatCvAUfKttLearuWrP9MDH5MBPbIqV92AaeXatLxBI9gBaebbnrfifHhDYfgasaacPC6xNi=xH8viVGI8Gi=hEeeu0xXdbba9frFj0xb9qqpG0dXdb9aspeI8k8fiI+fsY=rqGqVepae9pg0db9vqaiVgFr0xfr=xfr=xc9adbaqaaeGacaGaaiaabeqaaeqabiWaaaGcbaGaee4uamLaeeiuaaLaeyypa0tcfa4aaSaaaeaacqGGJaWicqWGubavcqWGqbauaeaacqGGJaWicqWGubavcqWGqbaucqGHRaWkcqGGJaWicqWGgbGrcqWGqbauaaaaaa@3A09@. A perfect clustering method would have SN = SP = 1, which implies that the false positive and false negative error are both zero.

## Authors' contributions

WR formulated the model, implemented the algorithm and performed the computational experiments. ArnoS suggested to use MRF and MFA, RK suggested data sets and provided biological evaluation. WR, RK and AS wrote the manuscript. AS supervised the work. All authors read and approved the final manuscript.
